# Mapping and Analysis of a Novel Genic Male Sterility Gene in Watermelon (*Citrullus lanatus*)

**DOI:** 10.3389/fpls.2021.639431

**Published:** 2021-09-01

**Authors:** Wei Dong, Dewei Wu, Chen Yan, Defeng Wu

**Affiliations:** ^1^School of Life Science, Henan University, Kaifeng, China; ^2^Jiangsu Provincial Key Laboratory of Crop Genetics and Physiology, Yangzhou University, Yangzhou, China

**Keywords:** watermelon (*Citrullus lanatus* L.), genic male sterility, fine mapping, pollen-specific leucine-rich repeat protein, seed production technology

## Abstract

Seed production is critical for watermelon production, which mostly involves first-generation hybrid varieties. However, watermelon hybrid seed production currently requires complex procedures, including artificial isolation and pollination. Therefore, the development and use of a male-sterile system to generate watermelon hybrids can simplify the process. The scarcity of male-sterile watermelon germplasm resources necessitates the use of molecular breeding methods. Unfortunately, the genes responsible for male sterility in watermelon have not been cloned. Thus, the genetic basis of the male sterility remains unknown. In this study, two DNA pools derived from male-sterile and normal plants in the F_2_ population were used for whole-genome resequencing. The Illumina high-throughput sequencing resulted in 62.99 Gbp clean reads, with a Q30 of 80% after filtering. On the basis of the SNP index association algorithm, eight candidate regions (0.32 Mb) related to specific traits were detected on chromosome 6. Expression pattern analyses and watermelon transformation studies generated preliminary evidence that *Cla006625* encodes a pollen-specific leucine-rich repeat protein (ClaPEX1) influencing the male sterility of watermelon. The identification and use of genic male sterility genes will promote watermelon male sterility research and lay the foundation for the efficient application of seed production technology.

## Introduction

Watermelon (*Citrullus lanatus*) is a monoecious and diclinous species, which makes it very useful for research applying heterosis for crop improvement. Heterosis generally refers to the phenomenon in which a few or many traits of hybrid varieties (heterozygotes) are superior to those of the parent varieties (homozygotes) (Gusmini and Wehner, 2005). A previous study revealed that the total fruit weight and total number of fruits were 22 and 32% greater in F_1_ hybrids than in the best parents (Gusmini and Wehner, 2005). Moreover, triploid watermelon breeding, which is based on the heterotic effects associated with different combinations of diploid and tetraploid genomes, requires the production of F_1_ hybrids (Saminathan et al., 2015).

Detasseling is a necessary step during the production of high-quality watermelon hybrid seeds. However, it is a time-consuming and labor-intensive process that is not conducive to plant growth, and it may lead to a decreased hybrid seed yield (Wan et al., 2019). This problem may be addressed by using male-sterile watermelon lines to generate hybrids. There are two main types of male sterility, namely, cytoplasmic male sterility (CMS) and genic male sterility (GMS). More specifically, CMS is maternally inherited and may be related to the genes in the mitochondria and chloroplasts of plants, whereas GMS is controlled by nuclear genes and leads to pollen abortion and stable sterility. Many male-sterile mutants have been identified among members of the family Cucurbitaceae, including watermelon, cucumber (*Cucumis sativus* L.), and melon (*Cucurbita pepo* L.). In cucumber, the genetic material of the natural male-sterile mutant plants in the “YL-5” inbred line population was sequenced, after which the *MS-3* gene was fine-mapped based on a Kompetitive Allele Specific PCR analysis (Han et al., 2018). The following five single recessive genes control male sterility in melon: *ms-1*, *ms-2*, *ms-3*, *ms-4*, and *ms-5* (Bohn and Whitaker, 1949; Bohn and Principe, 1964; McCreight and Elmstrom, 1984; Lecouviour et al., 1990; Pitrat, 2002; Park et al., 2004). These male sterility genes are on different chromosomes, and the male sterility traits controlled by these genes vary. A few male-sterile watermelon mutants have been reported, including a glabrous male-sterile (*gms*) mutant, male-sterile G17AB, a male-sterile dwarf (*ms-dw*), male-sterile *DT-2*, *ms-2*, and male/female-sterile *MFFH*, all of which exhibit GMS (Watts, 1962, 1967; Ray and Sherman, 1988; Xia et al., 1988; Dyutin and Sokolov, 1990; Zhang and Wang, 1990; Huang et al., 1998; Liu et al., 2004; Zhang et al., 2012). Male-sterile watermelon germplasm are very precious resources useful for watermelon breeding and for clarifying the molecular mechanism underlying male sterility (Rhee et al., 2015). However, the gene controlling male sterility has not been precisely identified and mapped.

Genic male sterility has been observed and widely used in various crops. For example, a Chinese hybrid rice line has been instrumental for increasing rice production in China and worldwide (Khush, 2001; Yuan, 2004). A spontaneous mutant exhibiting photoperiod-sensitive male sterility (PSMS) was detected in China in 1973. Specifically, PSMS was detected in the japonica rice (*Oryza sativa* ssp. *japonica*) variety Non-gken 58 (referred to as 58N) in Hubei Province, China (Shi, 1985). During hybrid rice breeding, PSMS rice may be fertile under short-day conditions, thereby enabling self-reproduction. It is male-sterile under long-day conditions and produces hybrid seeds by intercropping with normally fertile lines (Ding et al., 2012). Advances in genomic research have resulted in the mapping of many nuclear genes in rice (Qi et al., 2014; Sheng et al., 2015). A previous study revealed more than 70 male-sterile cytoplasmic systems in wheat and confirmed that heterosis may lead to yield increases of 3.5–15% (Singh et al., 2015). There are at least five GMS genes and three environmentally sensitive GMS genes (Ni et al., 2017). Hundreds of maize GMS mutants have been generated and characterized, and 17 maize GMS genes have been identified and cloned (Timofejeva et al., 2013; Walbot and Egger, 2016; Zhou et al., 2017). All of these genes are recessive, with the exception of *ms44* (Fox et al., 2017).

Next-generation GMS hybrid biotechnology-based research techniques are useful for screening GMS and maintainer seeds, especially the techniques related to the seed production technology (SPT) process previously used to address the problem associated with recessive genic male-sterile line maintenance and reproduction in maize (Albertsen et al., 2006). The male sterility of watermelon cannot be restored because of a lack of male sterility maintainer lines. Establishing SPT maintainer lines *via* the production of transgenic plants requires the following: the full coding sequence of a male fertility gene for fertility restoration, a pollen-inactivating gene for disrupting transgenic pollen development, and a seed color marker gene for seed sorting (Wu et al., 2016). The SPT process has been developed in rice (Chang et al., 2016; Wang et al., 2018). To date, there is no report describing the utility of SPT in watermelon. The G17AB male-sterile watermelon line was initially selected in 1988 and was subsequently used by watermelon breeding programs (Xia et al., 1988). It has been an excellent resource for constructing a biotechnology-based male-sterile system, but the major gene controlling the male sterility of G17AB remains unknown, which has hindered the development of SPT. The primary objective of this study was to identify the major gene regulating GMS. Bulked segregant analysis (BSA) and whole-genome resequencing of two DNA pools (i.e., male-sterile pool and normal pool) derived from the F_2_ population followed by genetics-, bioinformatics-, cytobiology-, and molecular biology-based analyses preliminarily identified *Cla006625* as a gene encoding a melon pollen-specific leucine-rich repeat (LRR) protein. Moreover, *Cla006625* was determined to be the major gene controlling watermelon male sterility. This study is the first to clone a male sterility gene in watermelon. The identification and characterization of the GMS gene provides the foundation for future research aimed at applying SPT for the genetic improvement of watermelon.

## Materials and Methods

### Plant Materials and Phenotyping for Male Sterility

The G17AB male-sterile watermelon line (2*n* = 2× = 22) was identified in 1997 at the experimental farm of the Henan University Genetics and Breeding Base in Kaifeng, China. Its morphological characteristics were documented annually starting in 1998 to evaluate its genetic stability. In this study, the following validation experiments were conducted. Firstly, G17AB male-sterile watermelon plants were tagged and hand-pollinated with pollen from G17AB male-fertile watermelon plants to generate the F_1_ generation in the spring of 2015. In the fall of 2015, the F_1_ plants were selfed to produce the F_2_ generation. Secondly, G17AB male-sterile watermelon plants were analyzed by conducting test cross experiments. The plants were hand-pollinated with pollen from six inbred lines (“Zhongliu,” “Cai1,” “Cai7,” “Kexi,” “Juwang,” and “Changhei”) to generate the F′_1_ generation in the spring of 2016. In the autumn of 2016, the F′_1_ plants were self-pollinated to generate the F′_2_ generation. The F′_2_ seeds were sown in the spring of 2017. The male flower fertility of the F_1_, F_2_, F′_1_, and F′_2_ plants was determined. In the autumn of 2017, 60 plants in the F_2_ segregating population (mixed pool comprising 30 plants exhibiting male sterility and 30 plants exhibiting male fertility) and six parent plants (male-sterile G17AB and “Zhongliu” inbred line) were used as research materials. We strictly controlled the watermelon growth conditions, with all plants cultivated under long-day conditions and examined in the greenhouse at the Henan University Genetic Breeding Base. The temperature was maintained at 25–30°C during the day and at 15–18°C at night. All watermelon seeds were stored in a seed storage cabinet at 4°C.

### Pollen Microspore Development

Buds of the G17AB watermelon plants were sampled every 2 days during the pre-flowering stage, when the flower buds were 2, 3–4, 5–10, and 10–20 mm long (Shan et al., 2016; Sheng et al., 2017). For the light microscopy examination, the anthers were fixed in a fixative solution (4% paraformaldehyde) for 24 h. They were then dehydrated, embedded in paraffin, and sliced as previously described (O’Brien et al., 1964). The slices were stained with toluidine blue and sealed in neutral gum after heating at 38°C. The samples were then observed using the ECLIPSE E100 microscope (Nikon, Tokyo, × 100 magnification).

For the transmission electron microscopy analysis, the collected anthers were immediately immersed in fixative solution (2.5% glutaraldehyde). The samples were post-fixed for 5 h in a 1% osmium tetroxide buffer solution, after which they were washed four times and dehydrated in a graded series (30, 50, 70, 80, 90, and 100%) of ethyl alcohol for 1 h. The samples were incubated at 37°C overnight and embedded in paraffin at 60°C for 48 h. Ultrathin sections (60–80 nm) were sliced with the DiATOME Ultra 45° knife (DIATOME Ltd., Biel, Switzerland) and stained with a 2% uranium acetate-saturated alcohol solution. The samples were examined using the HT7700 transmission electron microscope (Hitachi, Japan).

### Sample Collection and BSA Library Preparation

Total genomic DNA was extracted from the leaves of the parents and F_2_ plants after the flowering stage. Genomic DNA was purified from 60 plants in the F_2_ segregating population and five parent plants for a whole-genome resequencing performed by the Beijing Biomarker Technologies Corporation (Beijing, China). To generate bulked samples, equal amounts of DNA from each plant in the male-sterile (S-pool) and normal (N-pool) groups were mixed for a final concentration of 40 ng/μl. The DNA samples were sonicated to produce 350-bp fragments. After trimming the barcodes, high-quality reads were mapped to the *C. lanatus* 97,103 genome sequence (version 1^1^). The single nucleotide polymorphisms (SNPs) were filtered, including the loci with multiple genotypes, all supporting reads for SNPs with fewer than four reads, the loci with the same genotype between mixed pools, and the loci of recessive mixed pool genes not from recessive parents. An association analysis was performed to analyze the difference between the SNP indices of the two pools. The ΔSNP/indel index reflected the significant differences in the genotype frequencies between the two pools (Fekih et al., 2013; Hill et al., 2013). Candidate regions were extracted from the linkage group that exceeded the threshold (99th percentile). The Euclidean distance (ED) algorithm uses sequencing data to identify markers of significant differences between mixed pools, after which the regions associated with specific traits are evaluated (Hill et al., 2013). Theoretically, except for the differences in the loci related to the target traits, the loci tend to be consistent between the two pools. The results of the variation analysis and the BSA correlation analysis for the samples were plotted using Circos software^2^.

### Analysis of Candidate Gene Expression Levels

The *Cla009408*, *Cla006737*, *Cla006738*, *Cla001244*, *Cla006625*, *Cla009378*, *Cla009382*, *Cla007521*, and *Cla009410* expression patterns were examined by quantitative real-time (qRT)-PCR. The G17AB and “Zhongliu” plants were grown for about 60 days after sowing. Three replicates of the anther samples were collected, each comprising 10 anthers from individual plants. Total RNA was extracted and gene expression was analyzed as previously described (Dong et al., 2018). Briefly, total RNA was isolated from each sample using the TRIzol reagent (Invitrogen, Carlsbad, CA, United States). qRT-PCR was completed using TB Green^®^ Premix Ex Taq^TM^ II (Tli RNaseH Plus) and the Roche LightCycler 480 II instrument according to the manufacturer’s instructions. The PCR program was as follows: 95°C for 30 s; 40 cycles of 95°C for 5 s and 60°C for 30 s. The *ClYLS8* gene was used as an internal reference control. Primers were designed based on the *C. lanatus* 97,103 genome sequence using the Primer5 program. Details regarding the qRT-PCR primers used to analyze candidate gene expression are listed in Table 1.

**TABLE 1:** Sequence details for the qRT-PCR primers used to analyze watermelon candidate gene expression.

**Primer name**	**Forward primer sequence**	**Reverse primer sequence**
Cla009408	TATGTATCGCCGAATAATCCCGC	CATTGAATTGTTCTGACCCCTCA
Cla006737	GACTGTCCATATTGCATCCCTCTACC	AGCACCACTAATTTCTACGGCTTCCT
Cla006738	TCACTTCTCCACATGTCCTCCTCA	TTTGCTTGGCTCTAAAACCTTCCT
Cla006625	TCACTCATCGTCAACTTTTGGCTC	ATTGGCTGTCATGTTTTGAGGGTC
Cla009378	GGTGATTGTTGCTCTTCATTCTTG	GGGAGGCGTCGATTTTCTTAGT
Cla009382	CCACAAGCCTCAAAACAAGCGA	GGAATCTGGGAAAGAGAACCCC
Cla007521	GCCTCATCACTTCCCCTCACCTT	AATCCTTTAGCCTCAAAACCCTG
Cla009410	TCTTCCTCCCCCTTCCATCTTC	ACCCTTTTCACTTCGCCCACTC
ClYLS8	AGAACGGCTTGTGGTCATTC	GAGGCCAACACTTCATCCAT

### Single Nucleotide Polymorphism Analysis of Major Genes Controlling Male Sterility

To detect the SNPs in the major genes controlling male sterility, genomic DNA was extracted from the leaves of 30 male-fertile watermelon plants and 30 male-sterile watermelon plants according to the CTAB method (Murray and Thompson, 1980). The extracted genomic DNA was analyzed by agarose gel electrophoresis to assess the extent of any DNA degradation. The DNA concentration and purity were determined using the Nanophotometer spectrophotometer (IMPLEN, CA, United States). A forward primer (5′-ATGGCTTCTTTTCATAGAAAGCAAGCT-3′) and a reverse primer (5′-TTAATAGCCAGGAAACTGTGGTGG-3′) were designed to analyze the SNPs. The PCR products were sequenced.

### Bioinformatics Analysis

The homology of nucleotide sequences was analyzed online^3^. The Vector NTI Advance10 software was used to align the target gene sequence with the sequences of *Arabidopsis thaliana* homologs. A phylogenetic tree comprising the target gene and its homologous sequences was constructed using the MEGA 4.0 software (Kumar et al., 2001). A bootstrap analysis (1,000 replicates) was completed to assess the reliability of the tree (Felsenstein, 1992).

### Watermelon Transformation

The binary RNA interference (RNAi) vector DHpart27RNAi FADP1P4 was constructed as previously described (Li et al., 2019). Briefly, a 300-bp fragment of the candidate gene was inserted between the *Hin*dIII and *Xba*lI sites. The fragment was amplified by PCR using a forward primer (5′-CCCAAGCTTAACAATCGCTTCGTCGGCC-3′) and a reverse primer (5′-CTAGTCTAGAGGTTTCCTATCTCGGGTGGG-3′). The reverse sequence was inserted between the *Xho*I and *Kpn*I sites in the vector. The reverse complementary sequence was amplified by PCR using a forward primer (5′-CGGGGTACCAACAATCGCTTCGTCGGCC-3′) and a reverse primer (5′-CCGCTCGAGGGTTTCCTATCTCGGGTGGGAAA-3′). The resulting DHpart27RNAi FADP1P4-Y recombinant plasmid was inserted into the *Agrobacterium tumefaciens* strain GV3101 cells. The seeds of six inbred diploid “Kexi” lines were used as explant material to induce the development of adventitious shoots. Previously described methods were modified for the genetic transformation of watermelon (Yu et al., 2011; Liu et al., 2016; Ren et al., 2018). Briefly, watermelon seeds were dehusked and sterilized, after which they were sown on basic Murashige and Skoog solid medium and incubated for 2 days in darkness and then 2–3 days with a 16-h light/8-h dark photoperiod. The cotyledon explants were infected with the transformed *A. tumefaciens* strain GV3101 cells carrying the recombinant plasmid. The infected cotyledon explants were co-cultivated in darkness for 4 days. The timing of this step was considerably affected by the variety. The cotyledon explants were then transferred to selective induction medium and incubated for 4 weeks. They were then transferred to selective elongation medium and incubated for 2 weeks. The plantlets with well-developed roots were collected from the rooting medium and placed in plastic cups containing vermiculite.

## Results

### Morphological and Genetic Characteristics of Male-Sterile Watermelon

**FIGURE 1:**
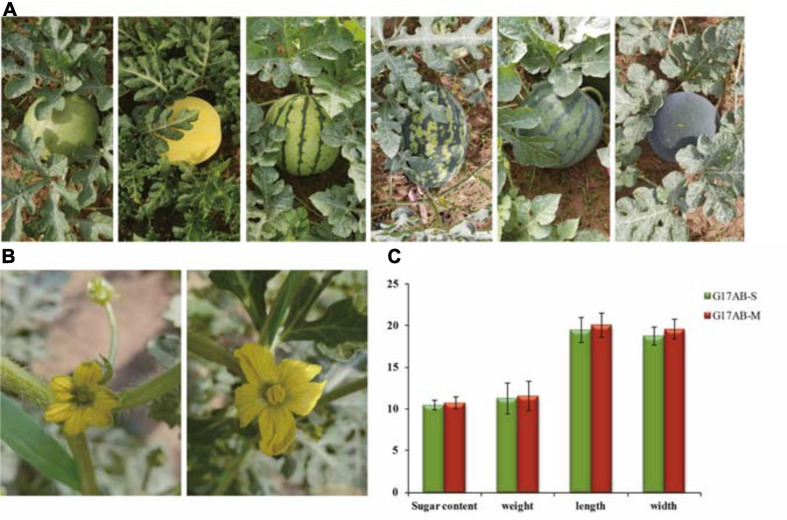
Comparison of the male flowers and fruits between male-sterile and male-fertile watermelon plants. **(A)** Different inbred lines (from *left* to *right*: “Cai1,” “Cai7,” “Kexi,” “Juwang,” “Tianli,” and “Changhei”). **(B)** Comparison of the flowers from male-sterile (*left*) and male-fertile (*right*) G17AB watermelon plants. **(C)** The sugar content, weight, length, and width of individual fruits from the male-fertile and male-sterile G17AB watermelon plants.

After years of screening and cultivating, the genetic characteristics underlying the male sterility of G17AB were confirmed to be stable. To evaluate the genetic stability, G17AB male-sterile watermelon plants were hand-pollinated using pollen from six inbred lines (“Cai1,” “Cai7,” “Kexi,” “Juwang,” “Tianli,” and “Changhei”) to produce F′_1_ and F′_2_ generations. The characterization of the resulting plants revealed that all F_1_ hybrids were male-fertile (Figure 1A), and the ratio of male-fertile plants to male-sterile plants in the F_2_ population was 3:1, which is consistent with Mendel’s segregation (the maximum χ^2^ value was 0.45, *P* > 0.05). The male-sterile flowers were pale yellow and had small petals and anthers, the surface of which lacked pollen grains. The male-fertile flowers were golden and had large petals and anthers, the surface of which was covered with pollen grains (Figure 1B). The average sugar content, weight, length, and width of the male-fertile watermelon fruits were 10.8°Bx, 5.8 kg, 20.1 cm, and 19.6 cm, respectively. The average sugar content, weight, length, and width of the male-sterile watermelon fruits were 10.5°Bx, 5.65 kg, 19.5 cm, and 18.8 cm, respectively (Figure 1C). There was no difference between the fruits produced by the male-fertile and male-sterile G17AB plants. The fruits were all round with red flesh and were moderately sized. The GMS mutant G17AB is an excellent resource for constructing a biotechnology-based male-sterile system.

### Microspore Development

**FIGURE 2:**
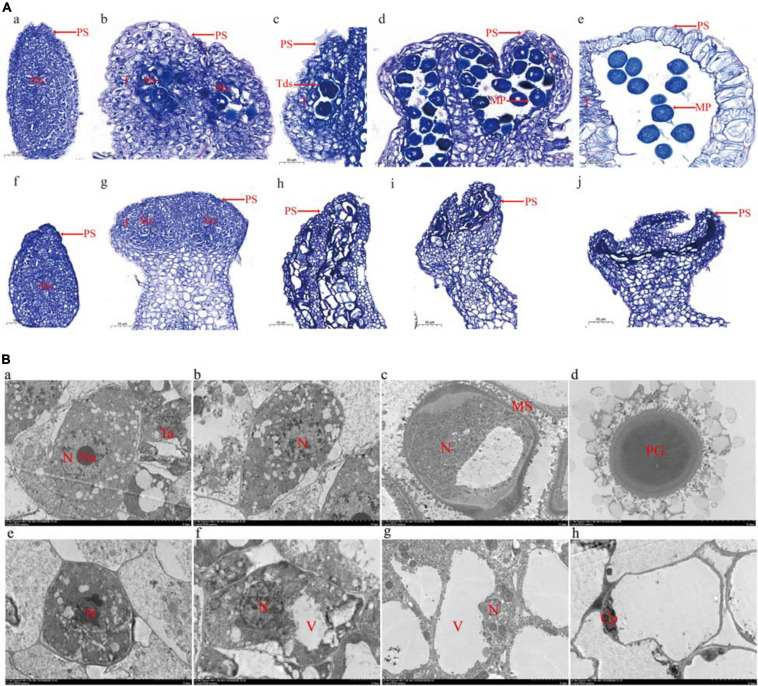
Microscopy analysis of microspore development. **(A)** Optical microscopy images. Undifferentiated anther **(a)**, microsporocyte stage **(b)**, tetrad stage **(c)**, microspore stage **(d)**, and mature pollen stage **(e)** in male-fertile anthers. Undifferentiated anther **(f)**, microsporocyte stage **(g)**, microsporocyte abnormal division stage **(h)**, microsporocyte disaggregated stage **(i)**, and withered powder chamber **(j)** in male-sterile anthers. *PS*, pollen sac; *T*, tapetum; *Tds*, tetrads; *Ms*, microsporocyte; *MP*, mature pollen. *Bar* = 50 μm. **(B)** Transmission electron microscopy images. Sporogenous cells **(a)**, microsporocyte **(b)**, immature pollen **(c)**, and mature pollen **(d)** in male-fertile anthers. Sporogenous cells **(e)**, microsporocyte **(f)**, abnormal microsporocyte **(g)**, and hollow and wrinkled pollen **(h)** in male-sterile anthers. *N*, nucleus; *Nu*, nucleolus; *Ta*, tapetum; *MS*, microspore; *PG*, pollen grain; *V*, vacuole; *Cp*, cytoplasm. *Bar* = 50 μm (each scale = 5 μm).

To analyze pollen development, anthers embedded in paraffin were examined using a light microscope (Figure 2). Normal pollen sacs were detected in the fertile flowers and the male-sterile flowers (Figure 2A, parts a and e). During the early male flower development stage, the pollen sacs of male-sterile flowers developed normally, similar to the pollen sacs of fertile flowers. The microsporocyte was larger in fertile anthers than in male-sterile anthers (Figure 2A, parts b and g). The archesporium was divided into a parietal cell and a sporogenous cell *via* periclinal division. The microsporocyte developed into a tetrad in the fertile anthers (Figure 2A, part c). In contrast, the male-sterile anthers lacked a tetrad and the microsporocyte was vacuolated (Figure 2A, part h). In the fertile anthers, the tetrad eventually developed into pollen (Figure 2A, parts d and e). In the male-sterile plants, the tapetum cells disintegrated, the anthers shrank completely, and no mature pollen grains developed (Figure 2A, parts i and j).

The transmission electron microscopy analysis revealed many organelles, including plastids, mitochondria, and an endoplasmic reticulum, in the cytoplasm of the microsporocyte in the fertile anthers. The nucleus was large and the nucleolus was clear (Figure 2B, part a). The mononuclear pollen grains formed by meiosis were filled with organelles (Figure 2B, parts b and c). The mature pollen grain wall comprised two layers (i.e., inner and outer walls). The outer wall exhibited protuberant ornamentation, and the pollen contained many plastids and starch grains (Figure 2B, part d). The transmission electron microscopy images clearly presented the boundary between the cytoplasm and the nuclear membrane. Compared with the microsporocyte in fertile anthers, the cytoplasm of the microsporocyte in sterile anthers was thinner and included fewer organelles (Figure 2B, part e). As the microsporocyte grew, the number of vacuoles in the cytoplasm increased rapidly, reflecting a tendency of vacuolation (Figure 2B, parts f and g). Finally, the vacuoles disappeared and the microsporocyte contracted into clusters and gradually disintegrated and disappeared (Figure 2B, part h).

### Whole-Genome Resequencing Analysis

**FIGURE 3:**
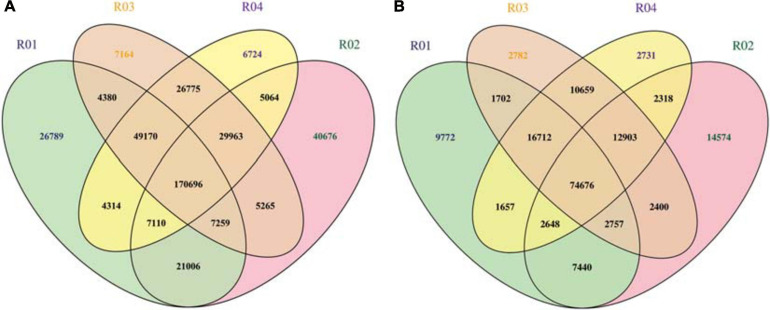
**(A)** Venn diagram of the SNPs among samples. **(B)** Venn diagram of the small indels among samples. *R01* represents the male parent sample; *R02* represents the female parent sample; *R03* represents the F_2_ generation male-fertile sample; *R04* represents the F_2_ generation male-sterile sample.

A whole-genome resequencing analysis was completed to identify and analyze candidate genes mediating male sterility. The Illumina high-throughput sequencing resulted in 62.99 Gbp clean bases, with a Q30 of 80% after filtering. The data have been submitted to the NCBI database (submission number: SUB8801923). The average comparison efficiency between the sample and the reference genomes was 98.55%, the average coverage depth was 34.25×, and the genome coverage was 99.60% (at least one base coverage). The SNPs between the watermelon sample and the reference genomes were obtained and are presented herein in a Venn diagram (Figure 3A). A total of 176,751 SNPs were detected between the parents, 2,852 of which were non-synonymous mutations. Additionally, 45,865 SNPs were detected between the mixed pools, 665 of which were non-synonymous mutations. On the basis of the mapping of the clean reads to the reference genome, the insertion and deletion of small fragments in the sample and reference genomes were detected (Figure 3B). A comparison of the parents revealed 72,356 small indels, whereas a comparison of the mixed pools detected 26,034 small indels.

**FIGURE 4:**
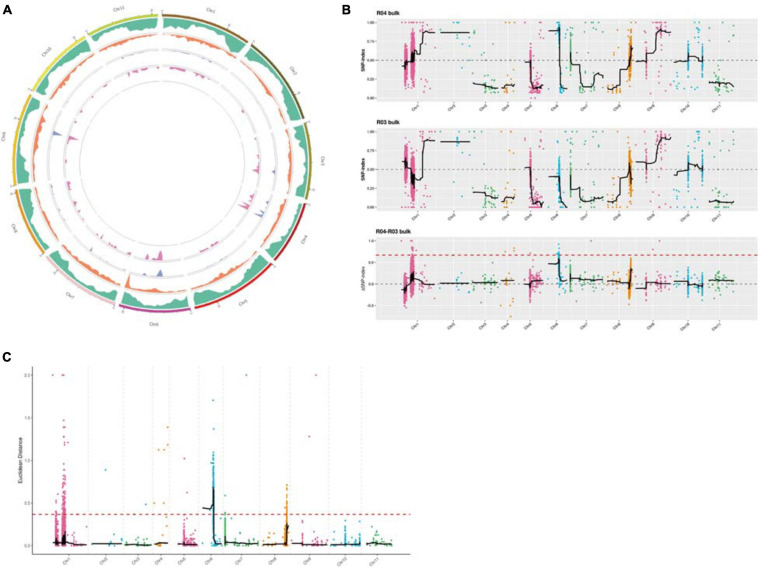
**(A)** Chromosomal distribution of the candidate regions determined using Circos software. From the *outside* to the *inside*: *first circle*: chromosomal coordinates; *second circle*: gene distribution; *third circle*: single nucleotide polymorphism (SNP) density distribution; *fourth circle*: Euclidean distance (ED) value distribution; *fifth circle*: ΔSNP index value distribution. **(B)** Layout of the ΔSNP index value distribution. The *x*-axis presents the watermelon chromosome numbers. The *colored dots* represent the calculated SNP index (or ΔSNP index) values. The *black lines* represent the fitted SNP index (or ΔSNP index) values. The *top figure* presents the SNP index value distribution in the male-sterile sample mixed pool. The *middle figure* presents the SNP index value distribution in the male-fertile sample mixed pool. The *bottom figure* presents the ΔSNP index value distribution, with the *red line* indicating the 99th percentile threshold. **(C)** ED value distribution. The threshold is indicated by a *red dashed line*. The *x*-axis presents the watermelon chromosome numbers.

A Circos plot of the chromosomal distribution of candidate regions among samples was produced (Figure 4A). On the basis of the theoretical separation ratio, the correlation threshold was 0.667 (Hill et al., 2013). To more precisely locate candidate regions, the threshold was decreased to 0.56. The candidate regions were extracted from the linkage group exceeding the threshold (99th percentile). Using the SNP index association algorithm, eight candidate regions (0.32 Mb) related to specific traits were detected on chromosome 6 (Figure 4B). The median + 3SD of the fitted values of all loci was used as the correlation threshold (0.37) of the analysis (Hill et al., 2013). The ED association algorithm revealed one candidate region (10.73 Mb) related to specific traits on chromosome 6. This region comprised 710 genes, including 25 genes with non-synonymous SNP sites (Figure 4C).

### Identification of the LRR Protein-Encoding Gene

**FIGURE 5:**
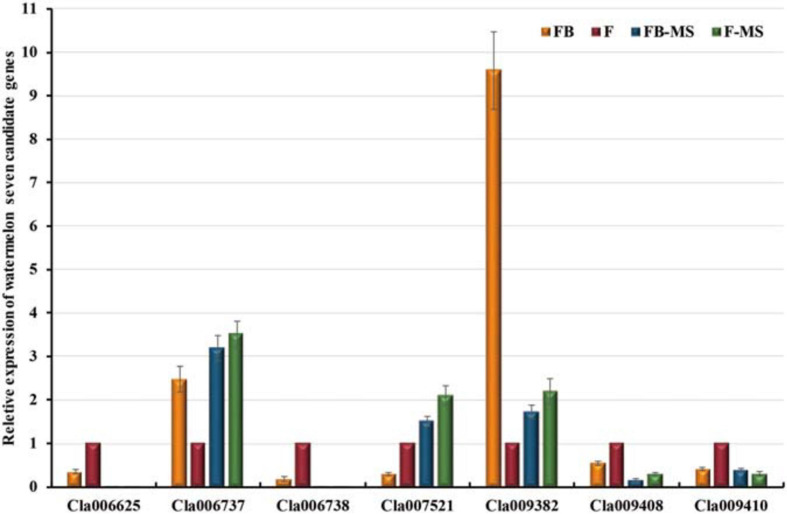
Relative expression levels of seven candidate watermelon genes. The presented data are the average values of six independent measurements. *The error bars* represent the standard deviation of the mean. *FB*, flower bud; *F*, flower; *FB-MS*, flower bud of male-sterile plants; *F-MS*, flower of male-sterile plants.

The expression levels of the following seven candidate watermelon genes in the “Zhongliu” and G17AB male-sterile plants were analyzed by qRT-PCR: *Cla009408*, *Cla006737*, *Cla006738*, *Cla006625*, *Cla009382*, *Cla007521*, and *Cla009410* (Figure 5). The *Cla006737* and *Cla009382* expression levels were higher in the flowers than in the flower buds of the G17AB male-sterile plants. The opposite expression pattern was detected in “Zhongliu” plants. The *Cla009410* expression level was lower in the flowers than in the flower buds of the G17AB male-sterile plants, whereas the opposite expression pattern was detected in “Zhongliu” plants. There were some inconsistencies between the candidate gene expression patterns and male sterility, which were initially excluded. The *Cla007521* gene, which is supposedly a suppressor gene, was more highly expressed in flowers than in flower buds. The *Cla006625*, *Cla006738*, and *Cla009408* expression levels were significantly higher in the flowers than in the flower buds of “Zhongliu” plants (*P* < 0.05). The expressions of the *Cla006625* and *Cla006738* genes were almost undetectable in the G17AB male-sterile line. These results indicated that the expression levels of these genes influence male sterility, implying that the genes may be responsible for male sterility in watermelon.

**FIGURE 6:**
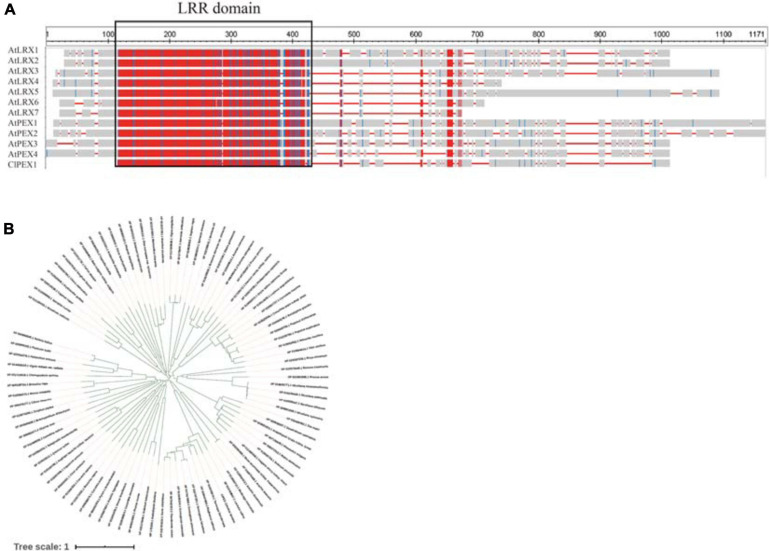
Characterization of ClaPEX1 and its predicted product. **(A)** BLAST analysis of the pollen-specific leucine-rich repeat extensin-like protein 3 sequences in various organisms. The GenBank accession numbers of *Arabidopsis thaliana* genes are as follows: AtLRX1 (AY026364), AtLRX2 (AAF70841), AtLRX3 (CAB40769), AtLRX4 (BAB01951), AtLRX5 (CAB37452), AtLRX6 (BAB01255), AtLRX7 (NP_197937), AtPEX1 (BAB01698), AtPEX2 (AAD43152), AtPEX3 (AAD41978), and AtPEX4 (CAA19879). **(B)** Phylogenetic tree based on pollen-specific leucine-rich repeat extensin-like protein 3 sequences in different organisms. The GenBank accession numbers are provided. The sequences are in Supplementary Material.

Alignments with sequences in the NCBI database indicated that *Cla007521* encodes a homocysteine *S*-methyltransferase, *Cla006738* encodes the MYB-related transcription factor LHY, *Cla009408* encodes a serine/threonine protein kinase, and *Cla006625* encodes a pollen-specific LRR extensin-like protein. The BLAST analysis suggested that *Cla006625* is the candidate gene most related to pollen. Consequently, it was preliminarily designated as a major gene controlling male sterility. The full-length *Cla006625* complementary DNA (cDNA) sequence consists of 2,076 bp. The SWISS-MODEL^4^ results indicated that the tertiary structure of the protein encoded by *Cla006625* is similar to that of pollen-specific LRR extensin-like protein 1. The sequence identities between *Cla006625* and *A. thaliana AtPEX1*, *AtPEX2*, *AtPEX3*, and *AtPEX4* were revealed to be 49.58, 56.31, 58.51, and 57.12%, respectively (Figure 6A). Thus, we speculated that *Cla006625* is a *PEX* homolog encoding pollen-specific LRR extensin-like protein 1 in watermelon. Accordingly, the gene was named *ClaPEX1* according to established gene-naming rules. The constructed phylogenetic tree clarified the molecular evolutionary relationship between *ClaPEX1* and its homologs (Figure 6B). The alignment of multiple amino acid sequences suggested that *ClaPEX1* is highly similar to pollen-specific *LRX* (*PEX*) genes in *Cucumis melo* (80.03% similarity; accession number: XP_008458675.1) and *Cucumis sativus* (76.37% similarity; accession number: XP_011656352.1). Additionally, *ClaPEX1* includes a pollen-specific LRR sequence, and the maximum homology was detected within this region, confirming that this gene encodes a pollen-specific LRR extensin-like protein.

To identify the mutation site in *Cla006625*, the SNPs in the gene were detected by sequencing the DNA extracted from the samples in the F_2_ population. Sixteen SNPs were identified in the *Cla006625* promoter and coding region in male-sterile F_2_ plants, of which nine were non-synonymous SNPs (Table 2). Multiple non-synonymous mutations to the gene likely influenced the watermelon traits.

**TABLE 2:** Chromosomal positions and the codons of 16 SNPs.

**Chr**	**Pos**	**Effect**	**Codon_change**
Chr6	2,366,479	Non-synonymous coding	aTc/aGc
Chr6	2,366,489	Synonymous coding	ctA/ctG
Chr6	2,366,498	Synonymous coding	tcT/tcA
Chr6	2,366,504	Synonymous coding	gaT/gaC
Chr6	2,366,529	Non-synonymous coding	Ggt/Agt
Chr6	2,366,539	Non-synonymous coding	gGa/gAa
Chr6	2,366,558	Synonymous coding	gaG/gaA
Chr6	2,366,614	Non-synonymous coding	aTc/aCc
Chr6	2,366,656	Non-synonymous coding	gAg/gGg
Chr6	2,366,669	Synonymous coding	gcC/gcT
Chr6	2,366,678	Synonymous coding	ttG/ttA
Chr6	2,366,686	Non-synonymous coding	aTc/aCc
Chr6	2,366,697	Non-synonymous coding	Ttc/Atc
Chr6	2,366,710	Non-synonymous coding	aAc/aCc
Chr6	2,366,725	Non-synonymous coding	aAt/aGt
Chr6	2,366,786	Synonymous coding	tcT/tcC

### Silencing of *ClaPEX1* in Watermelon *via* RNAi

**FIGURE 7:**
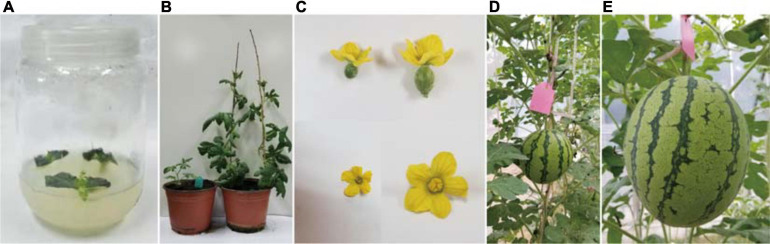
Characteristics of the transgenic watermelon plants at different growth stages. **(A)** Bud regeneration from cotyledons after 3 weeks. **(B)** Enlarged adventitious shoots, which were transferred to plastic cups containing vermiculite. The plastic cup on the *left* contains transformed plants, whereas the plastic cup on the *right* consists of control plants. **(C)** Elongated plantlets. Female and male flowers of transgenic plants are presented on the *left*, whereas female and male flowers of control plants are presented on the *right*. **(D)** Representative fruit from a T_4_ ClaPEX1 RNAi watermelon plant. **(E)** Representative fruit from a control “Kexi” plant.

To functionally characterize *ClaPEX1*, its cDNA sequence was amplified by PCR and integrated into the RNAi vector. The resulting recombinant plasmid was used to generate transgenic plants through *A. tumefaciens*-mediated transformation. To prove the universal utility of *ClaPEX1* RNAi, the seeds of six inbred diploid “Kexi” lines, which differ from the G17AB and “Zhongliu” lines, were used for adventitious shoot induction. Sixty transgenic plants were obtained and screened to verify that they were positive clones. After another 3 weeks, the regenerated shoots were transferred to the rooting medium (Figure 7A). Finally, the rooted shoots were grown in plastic cups containing vermiculite, ultimately resulting in 35 individual kanamycin-resistant plants after acclimation. After another 1 month, the transgenic plants were transferred to a greenhouse for further growth (Figure 7B). An examination 1 month later indicated that the flowers of the transgenic plants were blooming, and both the female and male flowers were smaller than those of “Kexi” plants (Figure 7C). Furthermore, very few fruits were produced by the transgenic plants following self-pollination. The fruits of T_4_ *ClaPEX1* RNAi watermelon plants exhibited a markedly inhibited seed set and were much smaller than the fruits of “Kexi” plants (Figures 7D,E).

## Discussion

Male sterility is a widespread phenomenon in higher plants. For crops with significant heterosis, male-sterile lines are important for generating hybrids. Therefore, there has been considerable interest in male sterility among breeders. Male-sterile germplasm resources for varieties with desirable characteristics are especially important. Genic male sterility has been exploited for the efficient production of F_1_ hybrid seeds because it can decrease production costs and enhance seed quality. Thus, GMS will likely continue to be applied for the production of agriculturally valuable seeds, with far-reaching implications for large-scale hybrid seed production. Moreover, GMS plants are useful experimental materials for investigating pollen and flower development, especially for watermelon, which currently has relatively few GMS materials. The male sterility of the watermelon mutant G17AB is controlled by a recessive nuclear gene. This line is an excellent resource for constructing a biotechnology-based male-sterile system. However, the genes responsible for male sterility have not been cloned and the genetic mechanism underlying male sterility remains unknown. In this study, we characterized the spontaneous GMS mutant G17AB and identified a candidate gene mediating sterility.

The genetic analysis conducted in this study revealed that a single recessive nuclear gene controls the male sterility of G17AB watermelon. Firstly, the F_1_ generation plants obtained *via* hybridizations with normal male-fertile watermelon plants were all male-fertile, whereas the ratio of male-fertile plants to male-sterile plants in the F_2_ population was 3:1, which was in accordance with Mendel’s segregation and the results of an earlier study (Xia et al., 1988). In the flowering stage, the male-sterile petals and anthers were small and pollen was undetectable on the anther surface. In contrast, the flowers of male-fertile watermelon plants had large anthers and a substantial abundance of pollen grains on the anther surface (Figure 1B). However, there were no differences between the fruits of the male-fertile and male-sterile G17AB plants. More specifically, all fruits were round, moderately sized, and had red flesh. In earlier investigations on cucumber, the four main types of male sterility were associated with undesirable traits (e.g., missing corollas, malformed ovaries, and unopened female flowers) (Grimbly, 1980; Zhang et al., 1994). Secondly, cytological examinations confirmed the presence of normal pollen sacs in fertile flowers, in contrast to the irregular pollen sacs in male-sterile flowers (Figure 2A). In a previous study, a rice male-sterile mutant (*OsMS-L*) was obtained following the ^60^Co γ-irradiation of an M_4_ population (Liu et al., 2005). Subsequent analysis of the tissue sections revealed the retarded tapetum development in the microspore stage of the male-sterile mutant. The tapetal cells expanded and the microspores degenerated. The tapetum degeneration retardation (*tdr*) rice (*O. sativa*) male-sterile mutant reportedly has a degenerated tapetum and middle layer as well as collapsed microspores (Li et al., 2006). In the current study, many cytoplasmic organelles were detected in fertile anthers during a transmission electron microscopy analysis (Figure 2B, part a). Additionally, as the microsporocyte grew, the number of vacuoles in the cytoplasm increased rapidly (Figure 2B, parts f and g). Finally, the vacuoles disappeared and the microsporocyte contracted into clusters and gradually disintegrated (Figure 2B, part h). These findings are consistent with the results of previous research. The anther abortion in the G17AB watermelon plants occurs during the meiosis of the pollen mother cell. An earlier investigation proved that the anther abortion of dicotyledonous plants commonly occurs from the sporogenesis period to the tetrad period (Liu et al., 2002).

Whole-genome resequencing and gene expression analyses identified *Cla006738*, *Cla009408*, *Cla007521*, and *Cla006625* as candidate genes, but the predicted functions of *Cla006738*, *Cla009408*, and *Cla007521* homologs were not associated with male sterility. Thus, of these genes, *Cla006625* is most likely the major gene contributing to male sterility. Because of the homology between the *Cla006625* sequence and the sequence encoding pollen-specific LRR extensin-like protein 1, *Cla006625* was renamed *ClaPEX1*. The LRR extensins (LRXs) are chimeric proteins that contain LRR and extensin domains (Baumberger et al., 2003). The full-length *ClaPEX1* cDNA is 2,076 bp long. On the basis of an alignment of multiple amino acid sequences, ClaPEX1 is most homologous to *C. melo* and *C. sativus* pollen-specific LRXs (PEXs). The sequence identities between *ClaPEX1* and the *A. thaliana* genes *AtPEX1*, *AtPEX2*, *AtPEX3*, and *AtPEX4* are 49.58, 56.31, 58.51, and 57.12%, respectively (Figure 6A).

The LRX-encoding genes can be divided into two groups based on their expression patterns. The genes in the first group are expressed in vegetative tissues, whereas the genes belonging to the second group are specifically expressed in pollen grains. For example, *TOML-4* in tomato (*Solanum lycopersicum*) and *LRX1–7* in *A. thaliana* are expressed in vegetative tissues (Zhou et al., 1992). In contrast, the gene encoding pollen extensin-like1 (*Pex1*) in maize and *LRX8–11* in *A. thaliana* are expressed exclusively in pollen grains (Rubinstein et al., 1995b; Baumberger et al., 2001). Thus, *ClaPEX1* likely belongs to the second group of LRX-encoding genes. Notably, *ClaPEX1* was substantially more highly expressed in the pollen of male-fertile G17AB plants than in the pollen of male-sterile G17AB plants. The *LRX8–11* genes in *A. thaliana* are reportedly expressed in mature pollen grains and possibly in germinating pollen grains, similar to the *ZmPEX1*, *ZmPEX2*, and *LePEX* genes (Rubinstein et al., 1995a,b; Stratford et al., 2001). The recently determined gene expression profiles of *A. thaliana LRX8*–*11* indicated that these genes are highly expressed in pollen tubes (Sede et al., 2018). In the current study, the T_4_ *ClaPEX1* RNAi watermelon fruits exhibited a markedly inhibited seed set and were much smaller than the fruits of the male-fertile watermelon plants (Figures 7D,E). In *A. thaliana*, the expression patterns and functions are consistent among *LRX8–11* in mature pollen grains and pollen tubes. Phenotypic analyses of mutants in which one of these genes is mutated confirmed that pollen germination and pollen tube growth are severely affected by the mutations, which significantly decrease the seed setting rate and the mutant allele transmission efficiency (Wang et al., 2018). Another study indicated that the main characteristics of an *O. sativa* LRR receptor-like protein kinase (RLK) single mutant are an increased number of microsporocytes and a lack of middle layers and tapetal cells (Yang et al., 2016). Very few pollen tubes make it through the papillar apoplast into the ovary, ultimately leading to male sterility and an inhibited seed set (Fabrice et al., 2018). Hence, *ClaPEX1* was identified as the most likely major gene causing male sterility in G17AB watermelon plants.

The identification and characterization of GMS genes has deepened our understanding of the molecular basis of anther and pollen development. This has enabled the development and efficient use of many biotechnology-based male-sterile systems for crop hybrid breeding. Next-generation GMS hybrid biotechnology will be useful for sorting GMS and maintainer crop seeds, and SPT will determine whether it can be successfully applied (Wu et al., 2016). In future studies, an SPT maintainer line will be developed *via* the transformation of plants with the *ClaPEX1* gene. The potential utility of the three other candidate genes identified in this study (*Cla007521*, *Cla006738*, and *Cla009408*) will be assessed in future investigations. The successful cultivation of a watermelon SPT maintainer line will improve the production of hybrid watermelon seeds by exploiting a homozygous male-sterile line. We generated preliminary evidence that *ClaPEX1* may be useful for generating an SPT maintainer line and for hybrid seed production.

## Data Availability Statement

The original contributions presented in the study are publicly available. This data can be found here: NCBI, BioProject accession PRJNA687916 (https://www.ncbi.nlm.nih.gov/bioproject/PRJNA687916).

## Author Contributions

WD and DFW jointly designed all experiments and conducted almost all of the molecular analyses. WD wrote the manuscript. WD and CY grew the plant materials and recorded the morphological characteristics. WD and DWW completed the whole-genome resequencing analysis. All authors contributed to the article and approved the submitted version.

## Conflict of Interest

The authors declare that the research was conducted in the absence of any commercial or financial relationships that could be construed as a potential conflict of interest.

## Publisher’s Note

All claims expressed in this article are solely those of the authors and do not necessarily represent those of their affiliated organizations, or those of the publisher, the editors and the reviewers. Any product that may be evaluated in this article, or claim that may be made by its manufacturer, is not guaranteed or endorsed by the publisher.

## Footnotes

1
www.icugi.org/pub/cucurbit/genome/watermelon/97103/v1/

2
http://circos.ca/

3
http://www.ncbi.nlm.nih.gov

4
https://swissmodel.expasy.org/interactive
