# Supplementary material for: Mapping and Analysis of a Novel Genic Male Sterility Gene in Watermelon (Citrullus lanatus)
Source: Front Plant Sci. 2021 Sep 1;12:639431. doi: 10.3389/fpls.2021.639431 (PMC8442748; doi:10.3389/fpls.2021.639431)
Supplement: Supplementary file 1 [file Data_Sheet_1.PDF]

>XP_014493019.1 [Vigna radiata var. radiata]
MASTSASCSFTISVLFLIFVHLSCLANSLNAKHSHNHRKPQHTENPPLNPRLHMAFLGLQA
WKQVIYSDPNNFTANWVGPSVCNYTGVYCAPSVDDPEVRVVAGIDLNFGDIAGFLPDEIGL
LSDLALLHLSNNRFCGILPMSLTNLTLLYEFDISSNRFVGPFPLVLLSLPMLTYLDLRYNEFEGP
LPPQLFNKTFDAIFLNNNRFSSSIPPNLGKNSASVLVFANNKFGGCLPESIVNFADTLEELVLI
NTSLSGCLPQQVGFLYKLRVLDVSFNSIVGPIPYSLAGLSHLEQLNLGHNMMSGTVPMGVC
ELPNLANFTFSYNFFCEEEGICQNLTSKRIVFDDRRNCLPEKPLQRSQKECSAKLEHPVDCSEL
CCVVGSNVSAGSVAVPPAAIPSAVPLSAPLLAPSHP
>XP_024197319.1 [Rosa chinensis]
MKSPNLTLSILIILSITFISKPSHQVSHSPQTFPVNARLQKAYQALQAWKHAITSDPNNFTAN
WCGPNVCSYTGVYCAQALDDPHITTVAGVDLNHANIAGSLPEELGLLVDIAVVHINSNKFC
GTIPSSFRGLYLLYELDISNNQFSGPFPSSVLCLPSLKYLDIRYNNFEGRIPPALFDLKLDALFLN
NNKFQCSLPQNIGNSSVSVIVLANNDFKGGFPSSLAKMKDTLNEVILTNSGLEGCLPSNIGC
LDKLTVFDVSNNKLDGSLPESMGGMKSLEQLNVANNGFSGQIPASVCSLHKLESFNSSNNY
FCGQPEMCVRLKETDDRRNCIPYRPLQRSAKECAAFHSHNNLANCGA
>XP_010669462.1 [Beta vulgaris subsp. vulgaris]
MTLPPRIFSNLLLLSLITFSLLHPSLARGHGNDDDNNNDNGLHKGWFKTKKSAESPEPTEAPE
SPESMLKSMESDGPEGVKVDPSYQFENDRLRKAYIGLQAWKNAMLSDPSNFTGNWVGPD
VCSYNGIFCAVLPTNNTSTTTSDPYDYNYTPPRVVASVDLNHADIAGYLPSELGLLTDLAVFH
INTNRFCGTIPKAFCSMKLLYELDVSNNRFVGKFPPALLGLPSLKYLDIRFNDFEGPIPPAIFDK
PLDALFLNNNRFRYGIPKNMGNSPVSVLVMANNDLGGCIPSSIGKMGYTLYELVLLNNNLT
GCLPEEIGNLKYVTVFDVSYNNLAGSLPSTISGMVSVEQLDVAHNRFTGVIPDAICQLPSLQN
FTYSYNYFTGEAPSCAALSGGEKVSNGSRNCIPGKKDQRSEHECSSPLAKPVDCSYYGCAKK
PTYSYSYSPPAYSGGASSGTYSPSPSYSSPQTPTTPYSPSPDYNTPATPSYTPSPVYNTPPTPS
YYTPSPDYNTPPTPSYYTPSPDYKSPSTPSYTPSPVYNTPPTPSYYSPSPDYNSPPNPSYTPSPI
YNTPPTPSYYTPSPDYKSPPTPSYTPSPVYNTPPTPSYYSPSPDYKSPPTPSYTPSPAYNTPPTP
SYYSPSPNYKTPPTPSSYSPSPDYKTPPTPSYYSPSPDYKTPPAPSSYSPSPDYKTPPTPSYYSP
SPDYKSPTPPSYYSPSPDYKTPPTPSYYSPSPNYYTPPSPPASYTPPSTPEYSTPPPSHHQYSSS
SPPPTPEYYSSPPPAYSSPSPIHYTPPTSSYLPPPPPSKADCTPGHEHSPPTPAYDNTPMPPVY
GVSYGSPPPPIYPGY
>XP_016747281.1 [Gossypium hirsutum]
MASRQSFQAFGCLTFFSCILFCSYAPTFALTDVEQSYIARRQLLALRENGELLDGYEYTVKTTE
KFENERLRRAFIALQAWKKAMYSDPKNITSNWFGPNVCDYKGVYCVQAMDDPKLKVVAGI
DLNHADIAGYLPVELGLLTDVALIHLNSNRFCGIVPESLSELTLMHEFDVSNNWFVGDFPKV
VLSWPSVKYIDLRFNNFEGCLPPELFEKDLDALFLNDNRFTCNIPETIGKSTVSVVTFANNKFE
GCIPRSIGKMSNLDEIIFSNNNLGGCFPQEIGLLRNVTIFDVSKNSFMGSLPANFSGFEKVDIL
DISGNKLTGSVPEDICKLPSLSSFKFSYNYFSEEHMACIKPERKNIVVEDAGNCVAGRMKQKT
DKECKQVVSNPVDCSKDKCTRGSPPKQNTPTPHRVSP
>XP_004505561.1 [Cicer arietinum]
MASLSSPHSFTSCMLFLFFLYLSCFFNNLNAKHDIANNHKHQHRHKHTQNPPSNPRLYRAF
LALQAWKHVIYSDPKNTTSNWIGPSVCNYTGIYCAPSLDDPKQTVVAGIDLNHADIAGFLPE
ELGLLCDLSLLHLNSNRFCGILPTTFTNLTLLYELDLSNNRFVGPFPSVVLSLPSLTYLDLRYNEF
EGPLPPQLFNKPFDAFFLNSNRFTSSIPRNLGQSKASVMVFANNKFGGCLPESIVNFADTLEE
LLLINTSLSGCLPQQVGFLYKLRVLDFSFNNIVGPIPYSLAGLSHLEQLNLGHNMMSGIVPMG
VCELPNLENFTFSYNFFCEEEGICKNLTSKRIVFDDRDNCLPEKPRQRSKKECSDNLERPVDCF

ELCCIEDGFGKNVTAGSVAIPPAAMPVSAPIVAPSFP
>XP_004242113.1 [Solanum lycopersicum]
MHHFSVHSSCSNTLFYPIIFFILFNAASTTNQISNYIDHIEISNSRKLLSYNGDVLAINPSFSIEN
FRLKNAYIALQAWKEVILSDPHNITQNWVGSNVCSYTGVFCSPSLDQPSELTVSGIDINHGD
IAGKLPHELGLLFDIGLIHINSNRFCGTIPESFLNLKLLFELDISNNRFVGKFPDVVVQMPNLKF
LDLRFNEFEGALPKQLFERDLDALFINNNRFSFELPDNFGNSPVSVMVLANNNFVGCVPVSI
GKMVRLNELLLLNNNFHSCLPNEIGMLKNVTVFDISYNEMMGSLPDSIGEMVSLEQLNLGH
NMFSGMVSSNICTLPNLENFVYEHNYFSEESPACLNLNSFADQQNCLRDRPMQRPALDCQ
RFLSNEVDCTTFKCALPSSSPLPTTPPENNCCICLPPPSPSPLPSIELPSSPLPLPAPSGSPSPPSI
SPSPSPSSPQPPDLPSPSPSPSFVEPQLPSPLPCENQRTPSSQDMSSPSP
>XP_011100435.1 [Sesamum indicum]
MIISYPSSLLPARILVVLLLVGYATSAHHHHNNHVSKNATNQILQQAYVALQAWKRVIYSDP
MNFTSNWDGPNVCNYTGVYCAPHLNDTNVLVVAGIDLNHADIAGFLPDELGLLSELALLHL
NSNRFCGILPPTLSNLSLLFELDISNNRFVGPFPSVVLSLPSLYYLDIRFNEFEGQLPPQLFNKKL
DAIFLNNNRFTSIIPPNLGPSTASVVVFANNGFGGCLPPSIAKFANTLEELLLINTSISGCLPTEV
GFLYKLRVLDVSFNQIVGPIPYSLAGLAHLELLNLAHNKLSGIVPDGVCVLPNLANFTFSYNFF
CEEEGICSNLTSKGIVFDDRRNCLPEKPLQRSKKECEAERPVDCFDHVCGKSTSSSAASYHIPL
SSTPPP
>XP_015692910.1 [Oryza brachyantha]
ALQTWKQTAIFSDPKNLTADWVGPGVCGYTGVFCAPLPGAPRGEVAVAGVDLNHGDIAG
YLPPELGMLTDLALLHLNSNRFCGLVPATLRRLRRLHELDLSNNRFVGGFPAVVLELPALRFL
DLRYNDFEGSVPPQLFDLPLDAIFLNHNRLRFELPDNFGNSPVSVIVLANNDFGGCLPASLG
NMSATLNEILLINNGLSSCVPPEVGMLREVTVFDVSFNSLAGPLPPEVAGMRKVEQLDVAH
NRLSGTVPEAVCGLPRLKNFTFSYNYFSGEPPSCARVVPAAGARRNCLPNRPAQRMPQQC
AAFYALPPVDCTAFQCKQFVPSPPLPPPPPPAYPGPLPPVYPIPYASPPPPPLYR
>XP_020406382.1 [Zea mays]
MHILRFLPLIIATLLSLLSLALTADEASSIAHDHDQLVAFEDELQPNDHVHIDIGIDIKINNPRLL
GAHKALQALKQALYSDPNNFTGNWVGPDVCAYNGVTCVPSLHNQSESAVASLDMNAAD
VAGYLPKDIGLMADLAVLHLNSNRFCGVIPEEIRNMTELYELDASNNRFVGPFPAAVLGIPKL
SYLDIRFNDFDGPIPPEVFLKPYDAIFLNNNRFTSGIPETVGKTKASVIVLANNQLGGCIPRSIG
EATTTLDQFIFTNNSLVGCLPVETGLLTNVTVFDVSDNALTGSIPPTLAGLSKVEQLDLSHNM
FTGDVPSHVCKLPALANLSVSYNFFTREASECSSTADENMDGTSFDDDANCLGQSRPMQR
GADECTPVVSKPVDCTKVQQCGWPSPPAVSSPPLLSSPPPVASSPPPRSSPPPPPIIIPPVRGT
KYQSPPPPLFPGY
>XP_008802123.1 [Phoenix dactylifera]
LTLSDAQATSIPRRELLTFHKTFVDLLNNLEFDISIDVDIRNPRLKQAYNALQAWKKAIFSDPT
NFTGNWVGHDVCSYNGIVCAPSLDDPSVLVVASVDFNGADLAGYIPIEIGLLNDTAILHINS
NRFCGVLPSSLSSLTLLHELDVSNNWLVGQFPSVVLDLPALKYLDLRFNNFEGSLPPKLFDKD
LDALFLNNNLFDSNIPENLGNSTVSVLVLANNKFEGFIPKTIGKMKSTLNEIILSNNGLIGSLPA
EIGSLSEVTVLDASFNSFSGVLPETFAGLKKLESLNIAHNMLSGIVPDSVCGIKSLGNFKLSDNY
FKGAASKCTSRSDLVFDDKGNCFLKWRQQKSRKACAPVLAQPIICHHLDGTECGISALAPAS
PKPLTRSPSPPSPMYSQVPPVQSPPPHSVYLPPKPLVQSPPLSIYSPPPPIYSPPPPLVYSLLPPV
YSSPPPLVYSPLPPIYSPPSLISFLPPPHSSPSPIFSPPPPVHLPPPPNIIISSPYLYNILPPVGGLNY
ASPPPPQYPGYN
>XP_022981727.1 [Cucurbita maxima]

MKGSGCCLFFSLLLLQSFSFFASALSDTEASFIARRQLLTFLETDDLPDDYESTVVVTETFPNSR
LRRAFIALQAWKRSIYSDPLNTTANWVGADVCSYTGVFCTSALDDPNVQVVAGIDLNHADI
AGYLPVELGLLSDIALFHINTNRFCGLVPPSFERMILLHELDVSNNRFVGPFPEVVLRIPNLKFL
DIRFNDFEGKLPPALFTKELDAIFLNNNRFTSHIPESFGDSPASVIVIANNNFTGCIPTSIGKMG
NTLNEVLMLNNALGGCIPGELSLLGNATIFDVSSNQLSGSLPMSLHGLKNVEIIDVSSNSLTG
VVHGGLCNLPKLAEFTFADNLFDGEDTRCVPQNRQDVWLDDADNCLQGRPKQKPAETCS
SEITKAVDCSRVKCGGGSTPPKQPEAPIDDPHRQSHVPMNHPPPPPVINSLPPPPPVASPPP
PPPVASPPPPPPVASPPPPPPS
>XP_016508047.1 [Nicotiana tabacum]
MQVYRRFFILLFSSILFSFSVALSDHEASLLARRQLSTLPENGKLPDNYEFEVHVNYTFPNSRLR
RAYIALKAWKEAVYSDPYKFTNNWKGPDVCKYKGVFCSPALDDPNVTVVAGIDLNHADIA
GYLPVELGLLTDAALFHLNSNRFCGIIPESFSRLRLMHELDLSNNRLVGPFPKVVLNLPNLKYL
DLRFNNFEGELPPQLFDKDLDALFLNDNRFVSTTPETIGNSSASVIVFANNKFHGCIPNSIGK
MSKLDEIVFMNNDLGGCLPVEVGLLKNVTVFDVAGNSFSGILPKTLNDLSHVEQLDLSHNTL
TGFVQENLCRLPNLKNFTFSFNYFNGEAKGCEPHMRKDVIFDDTNNCLPDRPKQKSQKQC
QPTVNKPIDCRKAKCGASSSKSPPHKEKEKEKKPLPPKPKPNHPTPKPSPKPQVYTPPPTQKK
ASPPPRLKSTPPPPVPPPPHVSSPPPPVHSPPPVHSPPPVHSQPPPPRLSPPPPFENVVLSPNI
GSIYASPPPPIFPGY
>XP_016567998.1 [Capsicum annuum]
MKFINLALCFFFFIIFFFSKPSFQASFEPNPRLFDAYIALQAWKHVITSDPRNFTKDWYGYNVC
NYTGVYCAPAPDNPNITTVAGIDLNHANISGYLPEDLGLLTDLAVFHINTNRFVGTIPRSFSKL
RILYELDVSNNLFCGQFPMAVLSLPSLKFLDIRYNQFEGKVPSSLWDRTLDALFINNNKFQFS
WPRNLGKSPVSALVMANIRVTGCIPSTIANMSKTLNEIILMNASLSGCLPQELGLLKNVKVFD
VSFNNLVGELPESIGGMKKLEQLNVAHNKFSGEVPASICSLPKLENFTYSYNYFCGEPKICLKL
KDKNDKKNCIPYRPSQRSASECKTFYSRGPVDCSSFGCRSRSPPPPPPPPPPPPPPPPKSHYY
HYP
>XP_021275697.1 [Herrania umbratica]
MKKKTHIKLHSSHLSLLLISLFIGFCSCEQHSISSHGGLTDQEVLYIKQRQLLYYRDEFGDRGER
VTVDPSLVFENPRLRNAYIALQAWKQAILSDPFNLTGNWVGSEVCSYTGVYCAPAPDNKKI
KTVAGIDLNHGDIAGYLPEELGLLTDLALFHINSNRFCGTVPHKFINLKILFELDLSNNRFAGKF
PDVVLKLPKLKFLDLRFNEFEGTVPKELFDKDLDAIFINHNRFRFNIPDNFGNSPVSVIVLANN
KFHGCVPSSLGNMSGLEEIILMNNGFRSCLPEEIGLLKNLTVFDVSFNKLMGPLPDKIGEMISL
EQLNVAHNMLSGKIPASICKLPKLQNFTFSYNFFTGEPPVCLSLQDFDDRRNCLPARPLQRS
AAQCKSFLSRPVDCSSFRCAPFVPSLPAPPPPSPPIPVPSPPVVVPSPS
>XP_015170456.1 [Solanum tuberosum]
MKLINLALSGFLFIIFFFSKPSFQASIKYEPNPRLFDAYIALQAWKHVMTSDPRNFTKDWYDY
NVCNYTGVYCAPAPDNPNITTVAGIDLNHANISGYLPEKLGLLTDLAVFHINSNRFEGSIPKS
FSKLRILFELDVSNNLFCGEFPSVVLSLPSLKFLDIRYNQFEGKVPSTLWDRTLDALFINNNKFQ
FSWPKNLGKSPVSALVMANINVTGCIPSSIAKMSKTLNEIILSNSSLSGCLPQELGLLKNVRVL
DVSFNKLVGELPESIGGMKKLEHLNVAHNKFSGEIPMSICSLPKLENFTYSYNYFCGEPKICLK
LKDRNDKKNCIPYRPSQRSESECKTFYSRGPVDCSSFGCRSPPPPPPPPPPP
>XP_006406541.2 [Eutrema salsugineum]
MTRKTMEKPYGCFLLLFFFIIHVLIHSGTALTDEEASFLTRRQLLALSENGDLPDDMEYEVDLD
LNFANNRLKRAYIALQAWKKAVYSDPFNTTANWFGPNVCSYKGVFCAPALDDQSIMVVA
GIDLNHADIAGYLPPELGLLTDVALFHVNSNRFCGVIPKSFSKLTLMYEFDVSNNRFVGPFPT

VALSWPSLKFLDIRYNDFEGKLPPEIFDKDLDAIFLNNNRFESIIPETIGKSTASVVTFAHNKFT
GCIPKTIGQMKNLNEIVFIGNNLGGCFPNEIGSLNNVTVFDASSNGFVGSLPSSLSGLANVEQ
MDFSYNKLTGFVSDSICKLPKLSNFTFSYNFLTERLRQVEAPTPTTHVVEAPEKSPSSSTLPPV
VTAPPPGNNDGGDDFILPPNIGFQYASPPPPMFPGY
>XP_019159041.1 [Ipomoea nil]
MASSLPFSSTASALVLLLLTLSSFKISSNAALHPRHPSTNATASPPRLQQAYVALQAWKKVIY
SDPHNITSNWEGTSVCNYTGIYCAPFPNDTSIQVVAGIDLNHADIAGFLPDELGLLTDLSLIH
LNSNRFCGILPETLSNLALLFELDLSNNRFVGPFPSVVLSLPSLKYLDLRYNEFEGQLPSELFSRS
LDAIFVNNNRFSSMIPSNLGSSSASVVVFANNFFGGCLPPSIANFANTLEELLLINTSLSGCLP
PEVGDLYKLRVLDVSHNDLIGPIPYSIAGLSHLEILNLGHNMFRGNVPEGVCDLPNLSNFTLS
YNFFCEEDGICSNLTSKGIMFDDRRNCLPEKPLQRSKKECEAAYEHPVDCLADGHCGA
>XP_015060729.1 [Solanum pennellii]
MQVYRCFLSLLLISAVLFSSSCALSDHEASLLARRQLSTLPENGNLPDNYELEVNVEYTFPNSR
LRRAYIALKSWKEAIYSDPSEFTSNWKGPDVCNYKGVFCSPALDDPNVTVVAGIDMNHADI
AGYFPVELGLLTDVALFHLNSNRFCGIVPESFSKLTLMHEFDVSNNRLVGPFPKVVLNMSSLK
YLDLRFNNFEGELPPQLFDKDLDALFLNDNRFVSTIPETLGNSSASVIVFANNKFHGCIPSSIG
KMSNLDEIVFMNNDIGGCLPVEVGLLKNVTVFNVAGNLLSGILPKTLDGLSHVEELDISHNTL
TGFVPENLCSSLSLKKFVFSFNYFNGEAKGCVARSRKDMSLDDTNNCLPGRAKQKSQKECQ
VIVNKRVDCSKAKCGGPSSKKNEVPPKPAPTPVPKPRLNPKSPPTLKKASPPPTQAVSSPPLV
HSPPPPVHSPPPPLVHSPPPRVLSPPVSSPPPLVHSPPPPPPPVQSPPPPQIHAPPPPVHSPPP
PPVHSPPPPPVHSPPPPQVHSPPPPVHSPPPPPIHSPPPPQVHSPPPPVHSPPPPPVHSPPPP
QVHSPPPPVHSPPPPVHSPPPPIVHSPPPPIHSPPPPVHSPPPPFKHVVLPPNIGSIYASPPPPI
FQGY
>XP_013466713.1 [Medicago truncatula]
MAHRYSNKALGCFILFSLLVPSFSTISFDGPQFPVVVSGEGDDPSKYEFGFTFDDTPAQSSDD
PAQAEAPTQLLDVMAPSQAPALSPTPLFSFDIGHSPASSPVIESGNGGNNNNNDDDNDHS
PASSSVIESGNGGNNNNNGGNNNNNDDSVTSFPATNFPNERLKHAYVAFQAWKDAIHS
DPFNTTGNWVGTDVCSYTGVFCAPALDDPKLNVVAGVDLTHADIAGHLPEELGLLKDAALF
HVNSNRFCGIIPESFKNLTLMHEFDISNNHFVGNFPSVVLTWPGLKYLDIRFNDFEGCLPHEL
FEKELDAIFLNNNRFTCGIPETLGKSKASVVTFANNQFTGCIPKSIGNMANLNEIVLLGNGLG
GCFPQELGMLGNIVVLDVSQNGFVGTLPNLSGLKNVEVIDITHNKLSGYVSNTICQLPMLTN
FTFSDNYFNGEAQTCVPSSNPNVVFDDANNCLPGRKDQKTSKECLPVLTKHVDCSQHCGG
GAPKQETPKVQPPLPPTLPPVEDIPPTRAPVEDIPPTLPPVEDIPPTRAPVEDIPPTRAPVEDIPP
TKAPVSSPPPPVHSPPPPVHSPPPPVHSPPPPVHSPPPPVHSPPPPVYSPPPPVHSPPPPPPV
HSPPPPVHSPPPSVNSPPPPTWDDIILPPHIGAEYRSPPPPTIEGY
>XP_024440759.1 [Populus trichocarpa]
MQASTCFLVICLLLLPSISSFSVALSHSEASFIARRQPLSLEKNVNLPDYFELKIELNITFPNPRLR
KAYIALQAWKKAIRSDPFNITGNWEGLRVCDYNGVFCAPALDDPKQNVVAGIDLNHFDIV
GYLPVELGLLTDIALFHINSNRFSGIIPKSFSRLTLLHELDVSNNRLVGPFPEVVISLPSLKYLDIR
YNDFEGGLPPEVFEKDLDALFLNNNRFTSTIPETLGSSPASVVVIANNKLTGCIPSSIGKMGST
LNEFVFLNNSLSGCLPSEIGKLGNATVLDVGSNSFSGVLPRCFKGLSQVERLDVSHNLLTGFV
PEGICKLPNLVNFTFSYNYFNGEAQACSPPKRKDITMDDTSNCLPDRPKQKSPKICHPVVSK
PVDCNKAMCGGSPSSSSLPKPPPQPSPSPKAHPPKVPKPAPCPPKLKLHPKPAPAPAPAPTD
DHNEESPISHSPSSPPPLVYSPPPPAYSSPPTAHLAPPLVHSPLPPVHSPPTAAPVPAPAPAD
NPSDKSPVDHFLSSPPPLIPSPPPPVHSPPPPIHSPPPPVHSPPPPVHSPPPPMVSPPPPPKVV

VPPNLGFSYSSPPPPTLPGY
>XP_025809255.1 [Panicum hallii]
MRKALLLLFVLCCLAAAGEALAAVGKEAASAELAVVVDPSWRFPNQRLRDAYVALQTWKQ
QAIFSDPRNFTADWVGPGVCNYTGVFCAPVPRGEPGAGELAVAGIDLNHGDIAGYLPSELG
LLTDLALLHLNSNRFCGLVPATFRRLRVLVELDLSNNRFVGAFPAVVLDLPALKFLDLRFNDFE
GAIPPELFDRPLDAIFLNHNRLHSQLPDNFGNSPASVIVLADNSFGGCLPASLGNMSDTLNEI
LLINNGLDSCVPPEVGLLREVTVFDVSFNALVGPLPQQVAGMRKVEQLDVAHNRLSGAVPE
AICALPRLKNLTISYNFFTGEPPSCARVVPPDGDRRNCLPNRPAQRTPQQCAAFYSQPPVDC
AAFQCKPFVPVPPMPPPPPAYPGPLPPVYPMPYASPPPPSHYR
>XP_021860234.1 [Spinacia oleracea]
MVSKTCTFVLVLYLLTLLKRLETKHDTHHSHPHHNHSQSSVSNNRLNQAYIALQAWKKVIY
SDPTNYTSNWVGSSVCSYRGIYCGPAINDNSTTVVSGIDLNHLDLAGFLPDEIGLLSDLALFH
INSNRFCGILPLTMSNLTLLYELDLSNNRFVGGFPSVVLSLPSLKYLDLRYNEFEGSVPSDLFN
NNSLDAIFVNNNRFTSVIPSNLGMSTASVVVFANNKFGGCLPPSIANFANSIEELLLINTSLTG
CLPVEVGYLYKLRVLDVSYNKLVGQIPYSIAGLAHLEQLNLAHNMMSGEVGDGICVLPNLA
NFTLSYNFFCEEQGICQNLTSRGVVFDDRRNCLPGKPLQRSNKACDAVLEHPVECEEFHCH
HS
>XP_010096273.1 [Morus notabilis]
MANYHHPFTNHIIAAFFGCFLFSPLFPTFSLALTDAEVSFIARRQLLTMPTENGDLPNDFEYEF
DLVITFANERLRKAYIGLQALKDAIYSDPYNFTGNWAGANVCAYNGVFCAPALDDPKLSVV
AGVDLNHADIAGHLPAELGLLTDVALFHLNSNRFCGIIPESFKKLVLMHEFDVSNNRFVGPF
PKVVLKWPSVKYLDLRYNDFEGEIPSELFVKQLDAIFLNNNRFKSKIPDSLGNSTVSVVTFAN
NKFNGCIPRTIGNMPNLNEIIFLNNELGGCFPPEIGLLGNMMVFDASDNDFVGTLPKSFTGL
KSLEEIDIANNKLTGFVSKSLCTLPKLANFTFSFNYFIKEDESCVPSSKSNVVFDDESNCLSGRP
KQKSARTCFPVVTKPVDCSKNCGGEGDSRASPPQPKPKPSPSPYHVPHSPSKPTSPSTPPPS
PSQKSTPPEPKLEPPTPSPDPSTPKSELPTKPPSPSPVEAPPKSHSPPSATPPTTPSQPSPSSPP
PHSPPKFPPAPVSATPPSPDPTDRHPVRGPSRPPPPAHSYSPPPQAPSTPQTPSHTPSAKSPP
PPSESDRSPVHVHRPPKSPPPPEQTHSPVPSPTPKKSPPPTEQPPSPSPSPKYEKSPPPPSPKY
VKSPPHSPENPSPPSPKYVKSPPQSPKTENPPSPSPKHVKSPPPHSPETENPTPPSPKHEKSPP
SPSPNHVKSPPPTPKTEHPPSPSPNYVKSPPPPTTEQPPVHYYSPPPPDSPVQSPPPPVHSPP
PPVFSPPPPVHSPPPPSPVYSPPPPVHSPPPPVYSPPPPVCSPPPPPVYSPPPLVPSPPPPSFD
DIVLPPTFGSQYSSPPPPVFQGY
>XP_012830948.1 [Erythranthe guttata]
DVCAYKGVFCEQALDDPTVTVVAGVDLNHADIAGHLPEEIGDLTDISLLHLNSNRFCGVIPES
IKKLKILYELDVSNNRFVGRFPEVVLSLPELKYLDLRYNDFEGELPSKLFDKDLDAIFLNSNRFRS
TIPENFGNSPASVVVIADNNITGCIPSSIGNMAGKLDEFIAADNILTGCLPEEITLLNTTTVFDV
SNNKLVGDLPRGLEYLQSLEIINIAKNKLRSKVPESVCSLPGLKNFTYSYNYFDNRDPECFRNA
FPGLSVEDEENCFANEADQRSDKECRSVLSEKVDCGAGCRRPTDGDSPKPTPPKGDSPNPK
GEPVITPSPKASPSPKTPKVSPSPKPTPSHRKQPKSDAPSPKASKTTPTPSPWSQPPKAAMPP
STGGGVPPKNVVPAPTKTQPQPQPSKGSVSPPTTAPPTKGSVPEGSSQAQAPAGTPPSTSK
APPTPAAPKSGGVPSPVQKPSKPSAPAPEPLNGVPPTGKSQPTPSFTPSPPPPAVFRSPVSSP
PPPSKNPSPSPSPTVKPPVSTPPPQMMEEELTSL
>XP_011460368.1 [Fragaria vesca subsp. vesca]
MKASGCILSFLFVFSSISSISLALTDAEASFLTRRQLLTLPEGGDLPENFENGVQLNMEFPNPRL
RRAYIALQAWKKAIYSDPLKTTANWVGPDVCHYKGVFCEKALDDPKLDVVASIDLNGADIA

GYLPAELGLLQELAVFHINSNRFCGIVPPSFCRMKLLYEFDISNNRFVGEFPSVYACLPSLTYLD
IRYNEFEGCLPAELFNKNLDAIFVNNNRFGCTIPETVGNSNVSVLVIANNNFEGCIPSSIGKM
RNLNELLFSNNKLGGCVPPELFQISNITVLDISSNSFSGIMSKSFKIDNVEEFSLANNQLTGFV
PESICKLPGLKNFTYSNNFFNGAAKECVPTSRNDIVMDDSSNCLPDRPKQKSSKECQAVVSK
PVDCSKAKCGGGGVPSKPPAVPQQPPKVETPKPPQTPHVQPPPVHSPPPAPVHSPPPPVKS
PPPASPPPAPVHSPPPPVQSPPPASPPPVAAPPPDDFVLPPNLGFQYQSPPPPMFPGY
>XP_002457818.2 [Sorghum bicolor]
MGTTLIRFMSLIVATLLLFPSSSLSLTTDEAAAIAHPQLAAFEEQPHPNDHVHIDIAIDIKINNP
RLLVAHRALHALKQALYSDPNNFTGNWVGPDVCAYNGVSCVPSLHNASESAVASLDMNA
ADVAGHLPKEIGLMSDLAVLHLNSNRFCGVIPEEITNMTELYELDASNNRFVGPFPAAVLGV
RKLSYLDIRFNDFDGPIPPELFLKPYDAIFLNNNRFTSGIPETIGKTKATVIVLANNQLGGCIPRS
IGEAAATLDQFIFINNSITGCLPVETGLLTNATVFDVSDNALTGSIPPTLAGLSKVEQLDLSRNR
FTGDVPSGVCKLPALANLSVSYNFFTSEAAECSSTADDGKSFHDDGNCMGQSRPMQRGA
DESGFGEDVNNLIGGGYQFFF
>XP_011030760.1 [Populus euphratica]
MQASTCFLVICLLLLSSFSSFSVALSHSEASFIARRQPVSLKKNVHLPDSFELKIELNITFPNPRL
RKAYIALQAWKKAIRSDPFNITGNWEGLRVCDYNGVFCAPALDDPKQNVVAGIDLNHFDIV
GYLPVELGLLTDIALFHINSNRFRGIIPKSFSRLTLLHELDVSNNRLVGPFPEVVISLPSLKYLDIR
YNDFEGGLPPEVFEKDLDALFLNNNRFTSTIPETLGSSPASVLVIANNKLTGCIPSSIGKMGST
LNEFVFLNNSLSGCLPSEIGKLGNATVLDVGSNSFSGVLPRCFKGLSQVERLDVSHNLLTGFV
PEGICKLPNLVNFTFSYNYFNGEAQACSPPKRKDITMDDTSNCLPDRPGQKSPKICHPVVSK
PVDCNKAMCGGSPSSSSLPKPPPQPSPSPKAHPPKVPKPAPCPPKIKLHPKPAPAPAPAPAP
TDDPHEESPISHSTSSPPPLVYSPPPPAYSAPPTAHLAPPLVHLPLPPVHSPPTAAPAPAPAPA
PADNPSDKSPVDHFLSSPPPLIPSTPPPAHSPPPPPPVHPPPPTVHSPPPPMVSPPPPLHCPP
PPMVSSPPPKVVVPPNLGFSYSSPPPPTFAGY
>XP_013628839.1 [Brassica oleracea var. oleracea]
MKNTTQSLLLLFFFFSFLLSVHSLSISSNAPLTDNEVRFIQRRQLLYYRDEFGDRGENVTVDPS
LVFENPRLRSAYIALQAWKQAILSDPNNITLNWIGSNVCSYTGVFCSRAPDNRRIRTVAGIDL
NHADIAGYLPEELGLLTDLALFHVNSNRFCGTVPHKFKHLKLLFELDLSNNRFAGKFPAVVLD
LPSLKFLDLRFNEFEGTVPKELFSKPLDAIFINHNRFRFELPDNFGDSPVSVVVLANNRFHGCI
PSSFVEMENLNEIIFMNNGLNSCLPADIGRLKNVTVFDVSFNELVGPLPESVGGMVSVEQLN
VAHNQLSGKIPASICQLPKLENFTYSYNFFTGEAPVCLRLPEFDDRRNCLPSRPAQRSKEQCA
AFLSRPPVDCESFKCGRSVTPLPPIVPQLPPPPPPSPPPPSPVYSPRPPPPVYSPPPPPPPVYSPP
PPPSVHYSSPPPPTVYYSSPPPPPPSPEYEGPLPPVIGVSYASPPPPPFY
>XP_020881815.1 [Arabidopsis lyrata subsp. lyrata]
MIKNKNGEALWLLLSSSFLSPYTVVATFDDEPSFPENADLTNDLEQKCFSVNKVDPNLKFEN
DRLKRAYIALQAWKKAIYSDPFKTTANWVGSDVCSYNGVYCAPALDDDSLTVVAGVDLNH
ADIAGHLPPELGLITDLALFHINSNRFCGIIPKSLSKLALMYEFDVSNNRFVGQFPEVSLSWPS
LKFLDLRYNEFEGSLPSEIFDKDLDAIFLNNNRFESVIPGTIGKSKASVVTFANNKFIGCIPKSIG
NMKNLNEIVFTGNNLTGCFPNEIGLLNNVTVFDASKNGFVGSLPTTLSGLASVEQLDLSHNK
LTGFVVDKFCKLPNLESFKFSYNFFNGEAESCIPGRNNGKQFDDTNNCLQNRPSQKPAKQC
LPVVSRPVDCSKDKCSGGSNGGSSPSPNPPRTSEPKPSKPEPVVPKPSESPKPEPQKPSKPQT
PKTPEQPSPIPQPPKHESPKPEEPENKPELPKQEESP
>XP_009801066.1 [Nicotiana sylvestris]
MQVYRRFFILLFSSILFSFSVALSDHEASLLARRQLSTLPENGKLPDNYEFEVHVNYTFPNSRLR

RAYIALKAWKEAVYSDPYKFTNNWKGPDVCKYKGVFCSPALDDPNVTVVAGIDLNHADIA
GYLPVELGLLTDAALFHLNSNRFCGIIPESFSRLRLMHELDLSNNRLVGPFPKVVLNLPNLKYL
DLRFNNFEGELPPQLFDKDLDALFLNDNRFVSTIPETIGNSSASVIVFANNKFHGCIPNSIGK
MSKLDEIVFMNNDLGGCLPVEVGLLKNVTVFDVAGNSFSGILPKTLNDLSHVEQLDLSHNTL
TGFVQENLCRLPNLKNFTFSFNYFNGEAKGCEPHMRKDVIFDDTNNCLPDRPKQKSQKQC
QPIVNKPIDCRKAKCGASSSKSPPHKEKEKEKKPLPPKPKPNHPTPKPSPKPQVYTPPPTQKK
ASPPPRLKSTPPPPVPPPPHVSSPPPPVHSPPPVHSQPPPPRLSPPPPFENVVLSPNIGSIYASP
PPPIFPGY
>XP_023525544.1 [Cucurbita pepo subsp. pepo]
MKGSGCCLFISLLLLQSFSFFASALSDAEASSIARRQLITFLETDDLPDDYESTVVVTETFPNSRL
RRAFIALQAWKRSIYSDPLNTTANWVGADVCSYTGVFCTSALDDPNVQVVAGIDLNHADI
AGYLPVELGLLSDIALFHINSNRFCGLIPPSFERMILLHELDVSNNRFVGPFPEVVLRIPNLKFL
DIRFNDFEGKLPHALFTKELDAIFLNNNRFTSHIPESFGDSPASVIVIANNNFTGCIPTSIGKMG
NTLNEVLMLNNALGGCIPGELALLGNATVFDVSSNQLSGSLPMSLDRLKNVKIIDVSSNSLT
GVVHGGVCNLPKLAEFTFADNLFDAEDNRCVPQNRQDVWLDDADNCLQDRPKQKPAEIC
SSEIRKAVDCSRVKCGGGSKPPKQPEAPIDDPHRQSHVPMNHPPPSPVINSLPPPPVASPPP
PVASPPPPVASPPPPVASPPPPVASPPPPVASPPPPPSPSPPPVSSPLAPLEDIILPPNLGFEYSS
PPPPLFPGY
>XP_010064140.1 [Eucalyptus grandis]
MQASRCCFSLALLLFSSYFTFSSCLSTSEASLIARRQLLALRENTKLPRNYEYVVEVNETFANSR
LRRAYIALRAWKEAMYSDPLNTTGNWVGPGVCEYNGVFCSPALDDPNLIVVAGIDLNHADI
AGYLPPELSLLGDLALFHINSNRFCGVIPESFEKLTLLYELDLSNNRFVGLFPKVILSLPQLKYLDI
RYNDFEGKLPPQLFEKDFDAIFLNNNRFTSTIPENFGNSPASVVVVAHNDLKGCIPRSIGKM
ANTLNELIFSNNGLAGCLPPEIGMLGNVTVLDVASNSFIGTLPKSFQGLSAIELLDISGNTMTG
FVPGGICGLPKLTNFTFSNNYFNGEDQACEPPSRRGIFLDDSSNCLPERPRQRPAKACVPVV
RKPVDCGRSRCGGPSRSSNPKPPASTPSPPKRKVHPPPTSPKSPPPKSTPPPPVRSPPPPVHS
PPPPVHSPLPPPVHSPPPPVRSPPPPPPIHSPPPPVHSPPPPPVHSPPPPPPPPVHSPPPPIKSLP
PPVQSPPLPVLSPPPPVHSPPPPLLSPPPPVHSPPPPPVFSPPPPPTPAFILPPEFGSRYASPPPP
AFPGY
>XP_015573449.1 [Ricinus communis]
MKNPYLTIALLGILTVFFSKPSYQASYSSSPPPPIPSPRLLKAYIALQAWKHAITSDPKNFTSN
WYGPDVCSYTGVYCAPALDDPHTITVAGIDLNHANIAGSLPEDLGLLTDLALFHLNSNRFCG
TIPDSFRHLRLLYEFDISNNQFSGELPPVLLCLTSLKFLDVRYNEFYGNVPSKLFDLKLDALFIN
NNKFKSSLPENFGNSPVSVIVLANNDISGCIPSSLTKMGRTLKQIILTNMGLNGCIQSDIGLLN
QVTVFDVSFNKLVGSLPDSMGEMKSLEQLNVAHNKLSGNIPESICLLPRLENFTYSDNYFCG
EPPVCLKLQAKDDRRNCIPHRPLQRSPEECKAFYAYPVNCNAFGCSRSPPPPPPPPPPPP
>XP_009352479.1 [Pyrus x bretschneideri]
MKKKTTTLFHLSLLTALFLATSSLFSGAAAQEHRRSSHIVSHGLLSDEEALYIKHRQLLYYRDEF
GDRGEHVTVDPSLVFENDRLRNAYIALQALKQAILSDPFNITGNWVGSNVCKYTGVFCTKA
LDNRTIRTVAGIDLNHGDIAGYLPEELGLLTDLALLHINSNRFCGTVPHKFNRLKLLFELDLSN
NRFAGKFPRVVLQLPKLKFLDLRFNEFEGTVPKELFDKDLDAIFINHNRFRFDLPDNFGNSPV
SVIVLANNKFHGCVPASLGNMSNLNEIILMNNGFRSCLPPEIGMLKNLTVFDVSFNQFLGSL
PETIGGMVSLEQLNVAHNLLSGKIPASICSLPSLQNFTYSYNFFTGEPPACLGLAGFDDKRNC
LPNRPAQRTAAQCKSFLSKPVDCSSFRCKPFVPSLPTPPPPSPPLPMPSPPPIVVPSTPPPVLP
QSPPPVYSPPPPPPPPPVLSPPPPPPPGFSPPPPPPVHYGSPPPSIVYESPPPPTPVYEGPLPPIF

GVSYASPPPPPFY
>XP_007042323.2 [Theobroma cacao]
MAKPQRLQAFGCFLFFSFLLSSLSTFTFALSDAEASYIAHRQLLTLPENGELPEDFEYEVKIVETF
ANQRLKRAYIALQAWKKAMYSDPLNTTGNWVGPNVCAYTGVFCAPALDDPKLSVVAGVD
LNHADIAGYLPAELGLMTDLALFHINSNRFCGIIPKSLSKLTLMHEFDVSNNRFVGPFPEVVLS
WPGLKYLDVRFNDFEGKLPCGIFEKDLDALFLNNNRFTSTIPETIGKSTVSVVTFANNKFSGCI
PHSIGKMANLNEITFMNNDLGGCFPAEVGLLSNMTVFDAGLNSFTGILPQSFSGLKKVELLD
ISHNKLTGIVPENVCKLSSLSNFTFSYNYFKGEANACIPPSRKDIVIDDTSNCLGGRPKQKSAK
ECYPVVSRPVDCSKDKCGGGSSPSKPHPPKTPSPYESSPSKPQPKPPVTAPPTPKPKPSTPEE
PHRRPPVQGHPKPPSSAPAPSPDPHDQSPVTPIRPPAPKNPTREPPPAPKNPTREPPPAPKK
PTPESPKSPAAPENPTPQPPKSSPMPSPDPHGNSPVGGVRSPPPPVYSPPPPVHSPPPPVYS
PPPPPVHSPPPPPVYSPPPPPPVYSPPPPVHSPPPPVYSPPPPPPVYSPSPPPPVHSPPPPVYS
PPPPVYSPPPPAHSPPPPPVYSPPPPPPPVHSPPPPLYSPPPPVRSPPPPLYSPPPPPVPTPSLP
PPPVASAPPPTEEFVLPPNLGFQYSSPPPPMFPGY
>XP_012436216.1 [Gossypium raimondii]
MGKPRRLQVFDFSFLFFCFLSSLSISSFALSDAEASYLSQRQLLTLPENGELPDGFEFQVQINLK
FDNSRLRRAYIALQAWKKAMYSDPKNITNNWVGPNVCGYSGVFCAQALDDPKINVVAGV
DLNHADIAGYLPAELGLMTDLALFHINSNRFCGIIPKSFSELTLMHEFDVSNNRFVGPFPDVT
LSWSSVRYIDIRFNNFEGQIPPELFEMKLDALFLNNNRFTSTISETIGQSTVSVVTFANNKFKG
CIPRSIGKMENLDEIIFMNNDLGGCFPPEVGSLKNLTVFDASLNSFVGALPQEFSNLIGVNVL
DISHNKLTGVLPENICKLPSLSNFSFSQNYFNGAANACVPTLRKDIVLDDTGNCLGDRPKQK
LGNECEAVLSHPIDCSKDKCGGGSSPKPPVKAAPKPQPKPPVHSPPQQVLSPPPPVYSPPPP
VLSPPPPVYSPPPQPVRSPPPPPVQSPPPPPPQPVLSPPPPVYSPPPPVQSPPPPVLSPPPPVS
FPPPPVQSPPPPTSPPVVALTPPPTKKVVLPPNLGFQYSSPPPPMFPGY
>XP_020521521.1 [Amborella trichopoda]
MVPSIREPICSAIIWWLLLVLTGGFCGISGHGFSRSEASSIELSHGHGHGFSASLHEALVDGPE
LEPGFGFRSHGLTFAEASSIKHRQLLYYIDRYGDRGESVFVDPSFEFENSRLREAYIALQAWK
MAIISDPMNITGNWIGPIVCNYTGVFCSKSLDGLNLTVVAGIDLNHSDIAGYLPDELGKLTDL
ALLHLNSNRFCGTIPHTFKRLKLLYELDLSNNRFAGRFPTVVLKLPTLKYLDLRYNEFEGPVPSS
LFNRPLDAIFLNHNRFHFEIPENFGNSPVSVVVLANNRFRGCIPSSLAKMAPTLNEIIIMNNG
LSGCVPEEFGALKNLTVLDVSFNKLVGNLPLNLGGLVSLEQLNVAHNMLSGQIPPQICSLPN
LDNFTFSYNFFEGEPPVCLRLPSFDDRRNCINGRPKQRPRKQCKSFLSHRVDCGSVKCGRVS
PSPPPVFSPPPPLVLSPPSPPVSLPPPVPYPPPPPHVHSPPPPSPVYSPPPPPPIHYSSPPPPPP
VPCNSPPPPPPMSELPPPYMGPLPPVTAISYSSPPPPPYY
>XP_006582471.2 [Glycine max]
MKICIKDVHIMSSEIVTKPLIWPLTCPSLPLNITLSNSFFSHLFYKSLGAFQGSTSHKMATSYFLL
LSLFLLSSSVSHALSNVEASFIVRRQLLHLHENDELSDNYADNYETNLTFPNPRLKSAYIALEAL
KKAIYSDPSNFTANWEGPNVCSYNGVFCEKALDDPKIDVVAGIDLNHADIAGYIPPEIGLLTD
LALFHINSNRFCGVLPKSFSKLKLLYELDISNNRFVGRFPEPVLLIPDIKFLDLRFNEFEGELPSEL
FNKSLDAIFLNNNRFTSTIPQNMGSSPASVMVFAYNNLTGCLPSSIGNMTKTLNEFVLINNN
LTGCLPAEIGKLEQVYVFDISQNNFVGMLPRTFDGLKNAEHLSIGHNKLTGFVPRNVCSLPN
LVNFTFSYNYFNGEEEGCVPPKKDVVLDDENNCIPNRPKQKVADVCNVVISKHVDCKCGSG
XPSHSSNPPTTPSSPSPKSPSESQPSSPPKVETPKPQPQPTPSTPKPQPSPTPSTPKILLSLFHL
VFFLLTLPPIPKPNSVFSSLYGTLSSGVLGVASPAIPHSASLFTLQFNPLVFASLIPSSKMLPIAC
>XP_019707248.1 [Elaeis guineensis]

MKKSGGSQHLHLLVLFLLSFAGSAFGFSSHGLTDAEAGLIRQRQLLYYRDEFGDRGESVTVD
PSLNFPNSHLRDAYIALQAWKLAIISDPLNLTANWVGSDVCSYTGVFCAPLPSDPHLIVVAGI
DLNHGDIAGYLPEELGLLTDLALFHINSNRFCGTIPHKFECLHLLFELDLSNNRFAGKFPDVVL
RLPSLKYLDIRFNEFEGGVPKALFDKDLDAIFINHNRFAFDIPDNLGNSPVSVIVLANNRFRGC
VPASLGNMSKTLNEIILLNNGLRSCLPPEIGLLKELTVFDVSFNQLVGPLPDSIAWMRSLEQLD
VAHNLLSGQIPAAICTLPHLQNFTFSYNFFTGEPPVCLKVPSFNDRRNCLPGRPAQRSAAQC
KSFLSHPVDCNSFRCAPFVPALPPPPPPSPPPPVYSPPPPSPPPPSPSPPPPSPPPPSPPPPPPS
PPPPS
>XP_022157690.1 [Momordica charantia]
MASLLRKQPLIACFLLLFLSLLSPSLSLTDTETAYIGRRQLLHLKEHDELPADFKLEIDIPDTFPNE
RLKKAYVALQAWKVAIYSDPENITATWQGADVCSYTGVFCAPALDDANINVVAGIDLNHY
DIAGYLPPELGLLTDLALFHINSNRFCGIIPTSFIKLILMFEFDISNNRFVGEFPEVVLEWPEAKYL
DLRFNDFEGELPPKLFTLEFDAIFLNNNRFTSTIPDTIGNSTVSVVSFAYNEFHGCIPSTVGNM
PNVNEMLFIGNNMSGCFPLELGNIANLTVLDVSNNGFVGKLPESLSETTKLEILDVSNNELTG
SVPVGICKLPKLDNFTFSYNYFDSADPTCMEDKNVNKIFDYDQNCLPNQPDQRDAEKCASV
VKKTVDCGSCGSGSPPTQDDSFDQSPSPRTRAPPPEQTIEAGAPPPEQTIEAGAPPPEQTIEA
>XP_010488556.2 [Camelina sativa]
MMKNTTTTTTHSLLLLLFFTLFSFSHSLSISSNASLSYNEVRFIQRRQLLYYRDEFGDRGENVT
VDPSLVFENPRLRSAYIALQAWKQAILSDPNNITLDWIGSNVCSYTGVFCSRALDNRRIRTV
AGIDLNHADIAGYLPEELGLLTDLALFHVNSNRFCGTVPRKFKQLKLLFELDLSNNRFAGKFP
TVVLQLPSLKFLDLRFNEFEGTVPKELFSKPLDAIFINHNRFRFELPGNLGDSPVSVIVLANNHF
HGCVPTSLVEMKNLNEIILMNNHLNSCLPADIGRLKNVTVFDVSFNELVGPLPKSVGGLVEV
EQLNVAHNLLSGKIPASVCQLPKLENFTYSYNFFTGEAPVCLRLSQFDDRRNCLPGRPAQRS
SRQCSAFLSRPPVDCRSFSCGRSVSQSPPFVALPPPPPPSPPLPPQVYSPPPSPPV
>XP_020974408.1 [Arachis ipaensis]
MKKENQNVSVVAVLTALLFAGTLSSVCASNSKQTSFSGELSHVETMYIKQRQLLYYRDEFGD
RGENVTVDPSLVFENQRIRNAYIALQAWKQAIVSDPLNFTANWVGSDVCSYTGVYCAPAL
DNPKIRTVAGIDLNHADIAGYLPEELGLLVDLALFHINSNRFCGTLPHRFDRLKLLFELDLSNN
RFAGKFPAVVLRLPTLKFLDLRFNEFEGGVPRELFDKDLDAIFINHNRFVFEIPDNLGNSPVSVI
VLANNRFHGCIPASIGNMSNLNEIILMNNELRACLPSEIGLLKNLTVFDISFNQLLGPLPDAIG
GAVSLEQLDVAHNLLSGNIPASICMLPHLQNFTYSYNFFTGEPPACLRLASFDDRTNCLPGR
PLQRSAAQCRSFLSKPVDCNSFGCKPFVPSPSHPSPPSSPSSPSPSPSSPPSIPSPSLPSPSSPP
STPSPSPPNAPGQAPPPSTPTPIPSPPRSEPSPIYSTATTSSTGPL
>XP_019052652.1 [Nelumbo nucifera]
MRSSGCFFLIFLLLFSSQDSFSSALSEAEVSFIAHRQLLSLPENGDLPENYEYQVDVNLTFPNA
RLRRAYIALQAWKEAIVSDPHKITTSWVGADVCDYKGVFCAPAPDDPKSTVVAGVDLNNA
DLAGFLPVELGLMSDLALFHVNSNRFCGIIPQSFSKMAVLYEFDVSNNRLVGPFPMPVTAM
PALKYLDLRYNDFEGSIPPELFDMDLDAVFLNNNRFQNEIPENLGNSPASVIVFANNRLTGCI
PHSIKKMENTLNEIIFTNNKLTGCLPMEIGSLENLTVFAAASNSLSGVLPKSFSGIKSVELIDISN
NFLTGFVPEGICKLPNLVNFTFSYNYFRGESPECESSRTKDVVVDDTDNCFPDRPKQKASKTC
TPVVNRPVDCGKSKCVRPSLPTPSRPHKPKPTPDVESSRNKN
>XP_012069688.1 [Jatropha curcas]
MEKSWHLSLALIWLVLSIVLFLSKPSYQDDIFPFPSFPILNPRLMKAYIALQAWKLAITSDPNN
FTANWYGHNVCNYTGVYCAPALDDPHILTVAGIDLNHANIAGYLPEELGLLKDLALFHLNS
NRFCGTIPASFIHLHLLYELDVSNNQFSGPFPYVVLYLPSLKYLDIRFNQFDGEIPEEVFDLPLD

ALFLNDNKFESSLPENLGNSPVSVFVLANSNVRGCIPPSLARMAATLEEIVLSNLGLTGCLRQ
DIGVLKGLKVLDLSFNKLSGYLPASIGEMRNLEQLNVAHNKFYGQVPQSICSLPNLKNFTYSF
NYFSGESPVCLRLPAIDDQRNCIPSRPFQRSPEECRSFYAHQRVICGANKCPKS
>XP_019414655.1 [Lupinus angustifolius]
MQASGCSVILSLLLVSSFSSFSFALSDVEASFIARRQLLHLHEHDELTEAYVENYITDLKFDNPR
LKRAYIAFEAWKKAIYSDPSNITSDWVGPDVCSYKGVFCAPALDDPKIEVVAGIDLNHADIA
GYIPPEFGLLTDLALFHINSNRFCGVLPKSFCNLKLMHELDISNNRFVGPFPQSVLCLQDIKYL
DLRFNDFEGELPSELFNKTLDAIFLNSNRFVSVIPENLGNSPASVIVLANNHFNGCLPGSIGQ
MDKTLNEFVLVHNNLSGCLPSEIGKLSSVEVFDVSHNMFVGVLPKTLNGLRKVEELSIANNK
LTGSVLHGICSLPSLVNFTFSYNYFNGEEEGCVPRSRKEIELNDERNCISDRPKQRDESECNAV
ISKPVDCNKAQCSHSSTPSHSNNPPSHTPSEPKPTPSTSNPPTETPSEPESSPQTPETPETQAP
PTPEMPKAQSPPTPEAPKAKPPPTPQTPKPEPPPTPKAEPPPKPQTPKPEPPPTPKSEPPPTPE
VEPPQAPKLQPPPTPETPKLSPPEEDPHREAPKGRLRTPPPPVQSPPPPTNSPPPPVHSPPPP
VHSPPPPVHSPPPPINSPPPPVHSPPPPVHSPPPPVHSPPPPVHSPPPPVQIWKNLHVHANL
Y
>XP_022763324.1 [Durio zibethinus]
MAKPQKLQAFGCFLFFFCFFSSLSTSTFALSDAEVSYIAHRQLLALPENGELPEDYEYEVKIIETF
ANQRLKRAYIGLQAWKKAMYSDPLNTTGNWVGPNVCAYTGVFCAPALDDPKLSVVAGVD
LNHADIAGYIPAELGLMTDLALFHINSNRFCGIIPNSLSKLTLMYEFDISNNRFVGPFPDVALS
WPGLKYLDVRFNDFEGKIPFGIFERNLDALFLNNNRFTSTIPETIGKSTVSVVTFANNKFQGCI
PHSIGKMANLNEIVFMNNDLGGCFPAEVGLLGNVTVFDASFNSFTGILPQSFSGLKKLDLLDI
SHNKLTGIVPENVCKLSSLSNFTFSHNYFKGEANACIPHSRRDVVLDDTSNCLAGRPKQKST
KECQPVLSRPVDCSKDKCAGGSSSPKPQPPSKSSPSKPQSKPQVTAPPTPKPQPKPPVTVPP
TPKTQSKPPVTAPTTPKPQPKRPVTAPPTPKPIPSTPDDPYGLSPVGGVRSPPPLVQSPPPLV
QSPSPPVHSPPPPPPVYSPPPPVNSPPPPVHSPPPPVYSPPPPVYSPPPPVHSPPPPVFSPPPP
VHSPPPPVFSPPPPVHSPPPPVFSPPPPVYSPPPPPPVYSPPPPIYSPPPPVYSPPPPVQSPPPP
VYSPPPPPRSPTPSPPPAVASTPPPTEEFVLPPNLGFQYSSPPPPMFPGY
>XP_015874268.1 [Ziziphus jujuba]
MANLLSKQAFGCFLLLLLLLIDSSFFFTFSSALTDAEASFIAQRQLLTLPKDGDLPDEFEYEFDLV
ITFANDRLKRAYIGLQALKKAIYSDPYNFTGNWVGANVCAYKGVFCAPALDDSNISVVAGL
DLNHGDIAGHLPAELALMTDVALFHLNSNRFCGIIPESFKRLKLMHEFDVSNNRLVGPFPKV
VLQWPEVKYLDLRYNEFEGKLPPELFVKQFDAIFLNNNRFRSTIPVTLGNSTASVVTFANNKF
NGCIPRSIGNMKNLNEIVFLNNELSGCFPSEIGLLEDVTVFDASNNGFVGTLPNSLSGLKSLEE
MDISKNKLTGFVSENVCKLPKLLNFSFGHNFFSGEAQACLPSFNMDTGLDDSGNCLPGRPN
QKSSKTCLPVVSKPVDCSKNCGGPSSSREPNPPKSSSPVSVPPKSPSPSPPPPMQSPPPPPPP
VYSPPPPPPVHSPPPPVYSPPPPPPVHSPPPPVYSPPPPPPVQSPPPPVHSPPPPVHSPPPPV
HSTPPPVHSPPPPVHSPPPPVRSPPSPVHSSPPPPTPVSFPPPPPPGGDDIVLPPTFGSQYSSP
PPPMFPGY
>XP_025687663.1 [Arachis hypogaea]
MGLLSSSYSSSFTTFALFLFLVQLSCFFNNLNAKHSVANSHNRRGHHHHNTQNPPLNPRL
DRAFLALQAWKSVIYSDPKNFTANWVGPRVCNYTGVYCAPSQDDPKVTVVAGIDLNHGDI
AGFLPAELGLLSDLALLHLNSNRFCGILPRTLSNLSLLHELDLSNNRFVGPFPTVVLSLPSLRYL
DIRFNEFEGSLPVDLFNKTFDAIFLNNNRLSSSIPRSFGRISASVLVFANNRFGGCLPESIVNLA
ETLEELVMINTSLQGCLPQQVGFLYKLRVFDVSFNKIVGPIPYSLAGLSHLEQLNLAHNMMT
GIVPMGVCELPNLANFTFSYNFFCEEEGICRNLTSKRTVFDDRRNCLPEKPLQRSKKECNAVN

QNPVDCIKLCCTTEGGFGNNATSSSSMPTMPISAPPFIAPSHP
>XP_024045790.1 [Citrus clementina]
MKVFGCFVLFFLVTCSSLSSICLALSDAEAAFITRRQLLTLPKDGKLPDNFDDEYGLKNKTFAN
ERLKKAYVALQAFKKSIYSDPFNTTANWVDNTDVCSYNGVFCAPALDDPNITVVAGIDLNG
NDIAGSFPAELGLLTDLALFHVNSNRFCGIIPDFSDWKLMFEFDVSNNRLVGSFPRVVLSWP
SLKFLDLRYNNFEGELPCDLFDMKLDALFLNNNRFSYSIPEKLGRSTVSVVTFAHNNFNGCIP
RSIGNMQNLNEIILSDNKLSGCFPSEIGSLKNLRVFDVSSNLFHGNVPQSFSSLESIQTLILSHN
QLTGFVSEQICKLPSLSNFTFSYNYFQGLGNECIPGSKVNSAFDDTSNCLAERPSQKWANTC
EPVVSNPVDCSRDMCSAGGTPPSIPYTAPPKPTTIPPAPELKTPTPAPPTPREAKVLRPKGTTP
PPVRAPWAPTPQPSKPSPSPPVQSPPIGVQYPPPSFLLHHQPPLPPVQSPSLPVQSAPPPSLR
HHHECNRQ
>XP_010530870.1 [Tarenaya hassleriana]
MAKPPPSLPSSFFLLGSLFLLFISIPSLALTDAEASFLAHRQLLALPENGELPDDLEFEVDLKVTF
ANSRLKRAYIALQAWKKAIYSDPFNTTGNWFGPHVCGYNGVFCAPALDDQSITVVAGVDL
NGADIAGHLPAEIGLMTDVALFHLNSNRFCGIIPKSFEKLTLMHEFDVSNNRFVGKFPSVVVS
WPAVKYIDIRYNDFEGEVPPELFKKDLDAIFLNNNRFTSTIPDSIGESSASVITFAHNKFTGCIP
KSVGNMKNLNEIIFMDNNLGGCFPSEIGKLANVTVFDASENSFTGVLPASFVGLTGVEEFDIS
GNKLTGFVLENICKLPNLANFTYSYNYFNGQADSCVPGGRKDIVLDDTRNCLPDRPKQRSA
KECAVVVNRPVDCSKDKCAGGGGAGSKPATPVKPENPHDESPVKQRRSPPPPPVYSPPPTV
YSPPPPVFSPPPPSPPPPSPPPPVHSPPPPPVYSPPPPPVYSPPPPVHSPPPPVYSPPPPPPVYS
PPPPPVYSPPPPPVHSPPPPVHSPPPPPVYSPPPPVYSPPPPPVQSPPPPPVYSPPPPPVYSPP
PPKPVQSPPPPVYSPPPPPKPVQSPPPPAQQVERPQPPPPSDEVIILPPFIGHKYASPPPPIFPG
Y
>XP_021911732.1 [Carica papaya]
MRATGCFFFLFLIFFSSFSSYFSLPPSNAEASFIAPRQLFTLLRDGDLPDNYVSTLNLNISFPNPR
LKGPYIALQAWKEAIYSDPFNTTKNWVGPDVCSYNGVFCAPALDDPNVNVVAGIDLNNAD
ISGHFPSELGLLTDLALFHVNSNRFCGILPKSFSNLKLLFELDVSNNRLVGPFPECVLSICSLRYL
DLRYNNFEGELPDKVFEKDFDAIFLNNNRFMSTIPETLGSSKASVIVFANNNFTGCIPSSIGK
MSSTLNEIVFSNNFLTGCLPPEIGMLANLTVFDIENNLFTGPLPKALAGLETVEELAIARNNLT
GFVVEELCSLISLKNFSFSHNYFNGEAEACEPRKRGDIDIDDSNNCLPDKTNQKSDKQCKAV
VSRPVDCSKSKCAKSSSSPNLKPQTPREQPIEPESPEKRSPPPPPPLVESPPPPLSSPPPPSPPL
VQSPPPPSRSLQPPSDPYPAAPPISENDVVILPPTIGFQYASPPPPIFPGY
>XP_009108764.1 [Brassica rapa]
MASVLAMAKTPSLGCCVFLLSFFFLSSSFVAYAISETEAAFLVRRQLLTLPENGELPNDIEYEVD
LKATFANSRLKKAYIALQAWKKAIYSDPFNTTGNWHGPHVCNYTGVICAPALDDPNVTVV
AGVDLNGADIAGHLPAELGLMTDVAMFHLNSNRFCGIIPNSFSKLTLMHEFDVSNNCFVGS
FPCVILTWPDVKYFDVRFNDFEGQVPPELFKKELDAIFLNNNRFTSTIPESLGDSTASVVTFAN
NKFTGCIPKSIGNMKSLNEIVFMDNGLGGCFPSEIGMLSNVTVFDASKNSFIGRLPTSFAGLT
GVEELDISGNKLTGLLADSICKLPNLVNFTYSYNYFNGQDGSCFPGGGRKETVLDDTRNCLP
DRPEQRSAQECAVVINRPVDCSKDKCAGGGSSTPSRPSLVPTVPVQKPSPVPSLPVPEPSPV
HKPQPPKESQQPDDPYDQSPVKNRRSPPPPAPVNSPSIPLPSPPLPPPVHSPPPPVNSPPPP
VHSPPPPVHSPPPPPPPPPVYSPPPPVFSPPPPPVNSPPPPVLSPPPPVHSPPPPVNSPPPPVFS
PPAHPPKSSSPPQTPSEPSPSPMIFSPQPSQSPPAVSSPPPGPPKVDCPPAAQAPAPSDELIT
PTPAPVENKQTPSAQAPAPSDELIIPPFVGHQYASPPPPMFEGY
>XP_020156799.1 [Aegilops tauschii subsp. tauschii]

MMRTAAVLLLLCLFAVAGSGGGVARAAAVWAEGIRATAAVEVDPAWRFPSPRLRDAYVAL
QTWKQQAIFSDPKNLTGNWVGPGVCGYTGVFCAPVPSSGELAVAGVDLNHGDIAGYLPSE
LGLLCDLALLHLNSNRFCGLVPDTFRRLVLLHELDLSNNRFVGAFPTVVLDLPSLRFLDLRFN
DFEGGVPSELFDRPFDAIFLNHNRLRFQLPDNFGNSPVSVIVLANNHFGGCLPASLGNMSD
TLNEILLINNGLTSCLPPEVGMLREVTVFDVSFNALAGPLPPEVARMQKVEQLDIAHNLLSGT
VPEAVCDLPRLKNFTFSYNFFTGEPPSCARVLPADGDRRNCLPNRPAQRMPQQCAAFYARP
PVDCAAFQCKPFVPPRPPPPPAYPGPLPPVYPMPYASPPPPPRYR
>XP_017439946.1 [Vigna angularis]
MRKKTYSFHFSLSLSLSLFVVVLLQSLCASEGISHNGPLTDAEATYIKQRQLLYYKDEFGDRGE
KVTVDPSFRFENDRLRNAYIALQAWKQAILSDPKNLTLNWVGPDVCNYTYVFCAQALDNP
KIRTVAGIDLNHGDIAGYLPEELGLLTDLALLHINTNRFCGTVPHKFDKLKLLFELDLSNNRFA
GKFPDVVLRLPQLKFLDLRFNEFEGTVPKELFDKDLDAIFINDNRFVFDLPDNFGNSPVSVIVL
ANNRFHGCVPAGIGNMKGLNEIILMNNAFRSCFPEEIGLLKNLTVFDVSFNQLLGPLPDAIG
GAVSLEQLNVAHNLLSGKIPESICKLPNLQNFTFSYNFFTGEPPRCLALPAADDRRNCLPARP
FQRSAGQCKSFLSHPVDCKSFRCKPFVPTLPPPPPPSPPLPSPPPSPVYVPRSPPPPPPVYSPP
PPPPVYSPPPPPPPVYSSPPPPSSPPVYYYNSPPPPPPSSPPPAPVYEGPLPPVIGVSYASPPPPP
FY
>XP_009390486.1 [Musa acuminata subsp. malaccensis]
MAPIKLFFFVPCLVFFLLSAAVAVLGVNEGEDETMEGGGVSLAVDPSFQFANSRLRDAYIALQ
TWKRTAIFSDPQNLTGNWVGPDVCSYFGVYCAASPDDPYLTVVAGVDLNHADLAGYLPKE
LGLLSDLALLHLNSNRFCGTVPPTFDRLRLLFELDLSNNRFVGKFPEVVLHLPALRYLDLRFND
FEGPIPPGIFERPLDAIFLNSNRLRAGIPATLGSSPVSVLVLANNDLGGCIPSSIGGMANTLNEII
LLNDNLTGCIPLEVGLLRRVTVFDVSFNRLQGPLPESVAGMQSAEQLDVAHNRLTGRIPPGV
CDLPSLQNFTYSYNFFTGQPPSCGRAGRAVAFDGKVNCIPGLPDQRSPKQCSSTAAHPFDC
RKSKCWSGGAVPSPYTKPPSPMRPPKYASRRRQLPPPPAPVGGKSRGYFKRASPPPPQYESS
PSTRSHPPPLPSYSPKQSPPPSPSGYHASLPPPAPPTHGYEPAKPPHIPSPPTTVSPPTQYYPT
PSPVPVQPPTYTSPPVAPPSQKPWQAPPPPAGYSSPPLPKYAPPPLEYSSSPPPPPPVVKPSP
LPHEHPSPLPSTPKQSPPPSLPPPSELPPPPAPPKRHEEPAPPILPPVVGVSYASPPPPVIPYY
>XP_021627281.1 [Manihot esculenta]
MTRLVRASGCFLLFTLFISFFSSSLFALTNDEASLIARRQLLSLLENEELPYGFEYKVDINVTFPN
QRLRRAYIALQAWKKAIYSDPFNTTSNWVGANVCSYKGVFCAPALDNRNLSVVAGIDING
ADIAGYLPPEMGLMTDVALFHINSNRICGIVPNSFSRLSLMYEFDISNNRFVGYFPKVVLAWP
NLKYLDIRFNNFEGCLPKQVFLKGLDALFLNNNRFRCNIPETIGNSTVSTVVFANNNFTGCIP
HSIGNMANLNEIIFMGNGLGGCFPPEIGKLKNATVFDVSFNQFAGNLPSSFADLKNVEELILA
NNKLIGVMPKNICKLPCLKNLNFSFNFFQGEDEACLQASKKDIVVDGDSNCMDNRQNQKS
LDTCLSILSNPINCSKDKCGGGGGEKPPSPPLVLSPPPLPPVPMQSPPQPPTPVQSPPPSVQS
SPPPVHSPPPPPPVHSPPPPIPSPPPPVLSPPPPVQSXPPPVHSPPPPVHSPPPPLVPSPPPPV
HSPPPPPPVYSPPPPTASPPPPLIISPPPADDVVLPPHIGFRYSSPPPPMFPGY
>XP_022930664.1 [Cucurbita moschata]
MASFPFPFLFSSSSLSSKALFLLLLHLSCLFKSLSAKHGVVVTHNDHHHHHHARSPSTNPRLH
QAFLALQAWKKVIYSDPKNHTTNWVGPSVCSYFGVYCAPSLNDSNVQVVAGIDLNHGDIA
GFLPNELGLLKDLSLLHLNSNRFCGVLPQSLANLSLLFELDLSNNRFVGPFPSVVLHLPNLTYL
DLRFNEFEGQIPPELFNKSLDAIFINSNRFTNIIPRNIGGKSASVIVFANNNLKGCLPPTIASFA
NSLEELLLINTSLSGCLPQEIGFLYKLKVLDVSFNELMGPLPYSLAGLGQLEQLNLAHNLFTGN
LFEGLCNLPNLENVTVSYNYFCEEEGVCRNLTAEGVAFDDRRNCVPEKPLQRSKKECSAVVE

RPVDCFEHPCGGGYGSSIAAAPASAPISATSSVVAPAHL

>XP_022864212.1 [Olea europaea var. sylvestris]
MANPSKMNAFGCFLVVFLSLFSSFSSALTDAEAAYIARRQLLHHPDDDSLPNESEYEIDARLT
FANPRLKKAYIALQAFKKAIYSDPQNFTANWEGANVCAYNGVFCAQALDNPKQSVVAGV
DLNHADIAGHLPIEIGHLVDISLLHLNSNRFCGIIPKSIAKLTLLFELDVSNNRFVGPFPEVVLEL
PSLKYLDLRYNDFEGQLPPQLFNKPLDALFLNDNRFHSTIPENFGNSTVSVLVLSNNKFHGCI
PKSIGKMANTLDEILLSNNALSGCLPEEITLLKSATVFDISDN
>XP_015954312.1 [Arachis duranensis]
MKKENQNVSVVAVLTALLFAGTLSSVCASHSKQTSFSGELSHVETMYIKQRQLLYYRDEFGD
RGENVTVDPSLVFENQRIRNAYIALQAWKQAIVSDPLNFTANWVGSDVCSYTGVYCAPAL
DNPKIRTVAGIDLNHADIAGYLPEELGLLVDLALFHINSNRFCGTLPHRFDKLKLLFELDLSNN
RFAGKFPAVVLRLPTLKFLDLRFNEFEGGVPRELFDKDLDAIFINHNRFVFEIPDNLGNSPVSVI
VLANNRFHGCIPASIGNMSNLNEIILMNNELRACLPSEIGLLKNLTVFDISFNQLLGPLPDAIG
GAVSLEQLDVAHNLLSGNIPASICMLPNLQNFTYSYNFFTGEPPACLRLASFDDRTNCLPGR
PLQRSAAQCRSFLSKPVDCNSFGCKPFVPSPSHPSPPSPASPPSPSSPSSPSPSPSSPPSIPSPS
LPSPSSPPSPPSPSSPNAPGQGPPPSTPTPIPSPPRSEPSPIYRPPSPPSSPPPPSPIYIPPSPTQP
APPSEPSPTPTPTPPYCVRSPPPPPPPPNSPSIPAPELSPPPPSPYHYTSPPPPTHSPPPPTYNQ
SPPPPPPCIEPPPPPPPSPTPYLPPPSPSPPPPPVQHNSPRPHHPPVHYSSPPPPPAQYSSPPP
PPAQYSSPPPPVHYSSPPPPTPVHYSSPPPPASSPPCEETPPSPTPSPPPSPVYEGPLPPVVGV
PYSSPPPPPFY
>XP_018447489.1 [Raphanus sativus]
MAKPPSFGCFFFLLFFFFLSSSFVAYAISDSEAAFLVRRQLLSLPENSEGELPEDIEYEVDLKATF
ANTRLKKAYIALQAWKKAIYSDPFNTTANWNGPHVCGYTGVVCAPALDDPNVTVVAGVD
LNGADIAGHLPVELGLMTDVAMFHLNSNRFCGIIPKSFEKMKLMHEFDVSSNCFVGPFPNVI
LSWPEAKYFDLRFNDFEGQVPPELFKKELDAIFLNNNRFTSVIPETLGESTASVVTFANNKFTG
CIPKSVGNMKNLNEIVFMDNGLGGCFPSEIGKLSNVTVFDASKNSFIGRLPTSFVGLTGVEEL
DISGNKLTGLVGDEICKLPNLVNFTYSYNYFNGQSGSCVPGGGRKEIVLDDTRNCLTDRPDQ
RSAQECAVVINRPVDCSKDKCAGGGGGSSTPSRPSPVHEPTPVVPTPVVEKPSPVPAVPVQ
KPQPPKESPQTDDPYNQSPVKNRRSPPPPHQSQPPVVSSPPPLPSPPPPVVHSPPPPVHSPP
PPVHSPPPPVHSPPPPVHSPPPPVYSPPPPPVYSPPPPPVFSPPPPVHSPPPPVQSPPPPVHS
PPPPVFSPPAQPPKSSSPPQTPSPAVVLPPPSQSPPVVYSPPPRPPQINSPPTPVEEKQMPPA
QAPAPVEEEQMPPAQAPAPVEKEDTPTAQAPAPVEKEQTPPAQAPAPVEEKQTPTSQAPA
PVEKEQAPPAQAPAPVEEKQTPTAQAPAPSDEFIIPPFIGHQYASPPPPMFEGY
>XP_015636072.1 [Oryza sativa Japonica Group]
MGTLPPFPPLLLLVAVIFSFLSSSCFAVTSHEAAAIARRQLLNFEKHGDHVHIDIDIEIKVSNPR
LAAAHRALHALKRALYSDPGNFTGDWAGPDVCAYNGVLCAPSPDNASASAVASLDMNA
ADVAGYLPREIGLLSDLAVLHLNSNRFCGVIPEEVANMTRLYELDVSNNRLVGAFPGAVLRV
PELSYLDIRFNDFDGPIPPELFLRPYDAIFLNNNRFTSGIPDTIGRSTASVIVLANNDLGGCIPPT
IGQAAATLDQFVFLNNSLTGCLPLESGLLANATVFDVSHNLLTGAIPATMGGLAKVEQLDLS
HNTFTGVVPGDVCGLPALTNLSVSYNFIAGEDAQCSSALLDAKLDKSLEDEANCMGNVRP
MQRSAGECAPVVSHPVDCSKTKPCGWPAPAKKPAPESSKHSPPPPPPPAPVQSPPPPAPVV
SPPSPVFSPPPAFSPPPPPKTSPPPPVSSPPPPPPPTMSPPPPIQEPVILPPILSAKYQSPPPPFFE
GY
>XP_007136648.1 [Phaseolus vulgaris]

MASGCFLLLSLFLFSSLSSSSYALSDVEASFIARRQLMHLNENEDLPSNYVSNYKTNFKFSNSR
LKSAYIALEAWKKAIYSDPSNFTSNWSGQDVCSYNGVFCEKALDDPKIEVVAGIDLNNADIA
GYIPPEVGLLVDLALLHINSNRFCGIIPHSFSNLKILYELDISNNRFVGQFPETVLSIPDLKFLDIR
FNDFEGELPSDLFEKSFDAIFLNNNRFTSTIPQNLGSTPATVVVMANNNLSGCVPASIGDMK
SLNEFILINNNLMGCLPTEIGNLEKVSVFDISNNNFVGVLPKTLNGLKNVEHLSISHNKFTGFV
PRNVCSLPHLMNFTFSDNYFNGEEEGCSPQKKEPALDDENNCIPDRPKQKQSNACNEVISK
PVDCSKDKCGHGSPPSSSPKTPPTHTPTPKQETPTPQQPKPSPPPEDNSHEEAPKHHERSPP
PPVHSPPPPVHSPPPPVHSPPPPVHSPPPPVHSPPPPVHSPPPPVHSPPPPPVHSPPPPVHS
PPPPVHSPPPPVHSPPHPSTLLLHPSTLLLPQFTLHHLLSIPHLPQFTLHHHQFTLLLPRFTLLR
HLFTLHHLQCTLHRHRCNLLHLLRLLLWKTSSFPLTSALLMLHPLHPSLPATRRVAHISTIYTSS
>XP_020698207.1 [Dendrobium catenatum]
MKREAIDPSPIAFLLFILALIPIAGHALTDAEAGYIRHRQLLYYRDGFSEKVSIPPSFSFPNPRLR
DAYIALQSWKLAIISDPKNKTGDWVGPDVCHYTGVFCAPLPSDPSLIVVASIDLNHGDIAGFL
PEELGLLCDLAVFHINSNRFCGTIPNSFDSMHLLFELDVSNNRFAGKFPDVVLRLPSLHYFDIR
FNEFDGEVPDALFEKKLDAIFINNNRFFDEIPDTVGSSPVSVLVLANNRFHGCVPSSLGNMS
DTLEEIIFLHNKLKTCLPKEIGLLKKVKVFDASFNSLVGELPEEIEGMVSLEELDVAHNYLTGEIP
EGICELPRLKNFTYSYNFFTGEPPACLNVPAFDDRRNCIKGRPKQRSARQCKAIPNEPVDCCS
FNCKPFVPAVPLPPPPSPPPPPSPPPPTPSPPPPPPSPPPPSPSPPPPPPPPPPPPPSPPPPSPS
P
>XP_019235430.1 [Nicotiana attenuata]
MQVYRRFFILLFSSILFSFSAGLSDHEVSLLARRQLSTLPESGVLPDNYEFEVHVKYTFPNSRLR
RAYIALKAWKEAVYSDPYKFTNNWKGPDVCKYKGVFCSPALDDPNVTVVAGIDLNHADIA
GYLPVELGLLTDAALFHLNSNRFCGIIPESFSRLRLMHELDLSNNRLVGPFPKVVLNMPNLKY
LDLRFNNFEGELPPQLFDKDLDALFLNDNRFVSTIPGTIGNSSASVIVFANNKFHGCIPNSIGK
MSNLDEIVFMNNDLGGCLPVEVGLLKNVTVFDVAGNSFSGILPKTLNDLSHVEQLDLSHNT
LTGFVPENLCRLPNLKNFTFSFNYFNGEAKGCEPNVRKDVFFDDTNNCFPDRPKQKSQKQC
QPIVSKPIDCSKAKCGASSSKSPPHKEKEKKPLPPKPKPNHPPPTPKTSPKPQVYTPPPTQKKA
SPPPPPLKSSPPPPVPSPPPPPLASSPSPQVFSPPPPVHSSPPPVHSPPPPVRSPPPPVHSPPP
PLVHSPPPPPIHSTPPPVHSPPPPPVHSPPPPPPVRSPPPPPPRLSSPPPFENVVLPPNIGSIYA
SPPPPIFPGY
>XP_017617698.1 [Gossypium arboreum]
MASRQSFQAFGCLTFFSCFLFCSYTPTFALTDVEQSYIARRQLLALRENGELPDGYEYTVKTTE
KFENERLRRAFIALQAWKKAMYSDPKNITSNWIGPNVCDYKGVYCVRALDDPKLKVVAGID
LNHADIAGYLPVELGLLTDVALIHLNSNRFCGIIPESLSELTLMHEFDVSNNRFVGAFPKVVLS
WPSVKYIDLRFNNFEGCLPHELFEKDLDALFLNDNRFTCNIPETIGKSSVSVVTFANNKFKGCI
PRSIGKMSNLDEIIFSNNNLGGCFPQEIGLLRNVTILDVSKNSFVGNLPANFSSFEKVDVLDIS
GNKLTGSIPEDICKLPSLSSFKFSYNYFNEEHMACTKPERKNIEVDDTGNCVAGRMKQKTDK
ECKQVVSNPVDCSKDKCTGGSPPSKPKSPPLPPVHSPPPPVHSPPPPVGLISYFLLLIPI
>NP_179188.1 [Arabidopsis thaliana]
MPHIYKQPLGIFQGFVPTLTDAEVSFIAQRQLLTLPENGELPDDIEYEVDLKVTFANHRLKRAY
IALQAWKKAVYSDPFNTTGNWHGPHVCGYTGVFCAPALDDPDVAVVAGVDLNGADIAG
HLPAELGLMTDVAMFHLNSNRFCGIIPKSFEKLSLMHEFDVSNNRFVGPFPSVVLSWPAVKF
IDVRYNDFEGQVPPELFKKDLDAIFLNNNRFTSTIPDSLGESSASVVTFAHNKFSGCIPRSIGN
MKNLNEIIFKDNSLGGCFPSEIGKLANVNVFDASMNSFTGVLPPSFVGLTSMEEFDISGNKLT
GFIPENICKLPKLVNLTYAYNYFNGQGDSCVPGSQKQIALDDTRNCLPDRPKQRSAKECAVV

ISRPVDCSKDKCAGGSSQATPSKSPSPVPTRPVHKPQPPKESPQPNDPYNQSPVKFRRSPPP
PQQPHHHVVHSPPPASSPPTSPPVHSTPSPVHKPQPPKESPQPNDPYDQSPVKFRRSPPPP
PVHSPPPPSPIHSPPPPPVYSPPPPPPVYSPPPPPPVYSPPPPPPVHSPPPPVHSPPPPVHSPP
PPVHSPPPPVHSPPPPVHSPPPPVYSPPPPPVHSPPPPVHSPPPPVHSPPPPVYSPPPPPPVH
SPPPPVFSPPPPVHSPPPPVYSPPPPVYSPPPPPVKSPPPPPVYSPPLLPPKMSSPPTQTPVNS
PPPRTPSQTVEAPPPSEEFIIPPFIGHQYASPPPPMFQGY
>XP_018838819.1 [Juglans regia]
MKKTTLFTHFDLSLLIVSFLGAVSVCAAERLLITPNGGLTDAEALYIRQRQLLYYRDEFGDRGE
NVYVDPSLVFENSRLRDAYIALQAWKQAILSDPFNLTSDWVGSSVCKYTGVFCAPAPDNPKI
RTVAGIDLNHGDIAGYLPEELGLLVDLALFHINSNRFCGTVPHKFKNLKLLFEFDISNNRFAGK
FPVVVLNLPQLKFLDLRFNEFEGTVPRELFDKPLDAIFINHNRFRFDLPDNFGNSPVSVIVLAD
NKFHGCIPASIGKMSNLNEIIMMNNGFRSCLPVEIGLLANLTVFDVSFNELLGPLPDSFGNLF
SIEQLNVAHNLLSGKIPAGICKLPNLQNFTFS
>XP_008224262.1 [Prunus mume]
MLSKMKNPSLTLSLGVIFSIIFLFKPSHQVSNPRLLQAYTALQAWKHVITSDPNNFTANWCG
LQVCNYTGVYCAPALDDPHTITVAGIDLNHANIAGSMPEELGLLTDLAIFHINSNRFCGTIPY
SFRYLRLLHELDISNNQFSGQFPSFVLYINSLKYLDIRYNNFHGEVPSALFNLKLDALFLNNNR
FQFSLPQNIGNSSLSVIVLANNDLKGCIPSSLANLKDTLNEVILINSGLKGCLPSNLGLLDKVR
VFDISQNKLVGALPESMGGMKNLEQLNVAHNKLSGEVPASICLLPKLENFTHSYNYFCGEPP
ICIKLPEQDDRKNCIAYRPLQRSPQECATFYAHLVNCDAFGCTPRSPPPPSPPPPPPHVASHY
P
>XP_021715918.1 [Chenopodium quinoa]
MGSQRSKTSFLLLVLSLFSLLHKLETKHNTHHSHPHHNHKNSQSSAANPKLNQAYIALQAW
KKVIYSDPTNYTSNWVGPSVCRYRGIYCGPAIDDNTTTVVSGIDLNHLDLAGFLPDEIGLLAD
LALLHINSNRFCGVLPLTLSNLTLLYELDLSNNRFVGPFPSVVLSLPSLKYLDLRYNEFEGPVPS
ELFNNKSLDAIFVNNNRFTSVIPSNLGMSTASVVVFANNKFGGCLPPSIANFANSIEELLLINT
SLTGCLPEEVGYLYKLRVLDVSYNQLVGRIPYSIAGLAHLEQLNFAHNMMSGEVSDGICVLP
NLTNFTLSYNFFCEEQGLCGNLTSKGVVFDDRRNCLPGKKLQRSKKVCDVILEHPVECEEFN
CDYS
>XP_021691956.1 [Hevea brasiliensis]
MRHHSYLTLALWGILIFFSKPSYQASYSPSPPILNPRLLDAFIALQAWKHSIISDPKNFTSNWY
GPNVCNYTGVYCAPAPDDPHTTTVAGIDLNHANISGYLPEKLGLLTDLALFHLNSNRFCGTI
PDSFRHLRLLYELDISNNQFSGEFPYIVLYLPSLKFLDIRYNEFHGNVPSKLFDINLDALFINNN
KFNSSLPDNFANSPVSVVVLANNNIGGCIPSNLAKMAGTLSQILLMNMGLTGCLQSDIGLL
NQVKVFDVSFNKLVGTLPDSIGEMKMLEQLNVAHNKLSGEIPESICLLPKLENFTYSYNYFCS
EPHVCLKLSAKDDRKNCIPYRPLQRSPEECKAFYAYPVKCDAFGRCSPRSPPPPPPPPPPPPP
PPPPPPPPPPP
>XP_023923612.1 [Quercus suber]
MASSLVSSLTTRAIVLLLVNISFSFNNLAAKHRHHATHHHRFHSHSPTSNPRLHQAYIALQA
WKKVIYSDPKNFTTNWVGPSVCSYTGVFCAPALDDPEIRVVAGIDLNSADIAGFLPNEIGLLC
DLALIHLNSNRFCGTIPETLANLTLLYELDLSNNRFVGPFPSVVIPLPTLRFLDIRFNEFEGPLPP
ELFNKELDAIFVNNNRFTKMIPPNLGLSTASVVVFANNRFGGCIPPSISNFANSLEELVLINNN
LTGCLPQELGYLYKLRVLDVSSNSIVGPIPYSLAGLAHLELLNLGHNMLSGIVSEGVCLLPSLK
NLTFSYNFFCEEQGICQNLTSRKGLKYDDRQNCLPEKPMQRSAKECNATLEHPVDCFEHPC
TGAGGGGAKTAFGTAFIPAAAPAIVPVSSPFIPPSYT

>XP_006476177.1 [Citrus sinensis]
MRPKINIILPLFAILTLITLVTAEKLSVSNNGVFLEAEAAFIKQRQLLYYRNRYGGRGEFGRIPPS
FKFENSRIRSAYIALQAWKKAIISDPWNLTSNWVGCYVCNYTGVFCAQALDNHSIRTVAGID
LNHGDIASYLPEELGLLADIALFHVNTNRFCGTLPRSFNKLKLLFELDLSNNRFAGKFPYVVLG
LPKLKFLDLRYNEFEGKIPKALFDKDLDAVFINNNRFSFELPDNIGNSPVSVLVLANNKFHGCL
PLSFGNMSRTLNEVILTNNGLHSCLPEEIGLLKNVTVFDVSYNKLMGELPDTIAEMTSLQQLN
VAHNMLSGTVPDSVCSLPNLRNFSFDYNFFTGESPVCLDLQGFDDKRNCLRDKPRQRSTLQ
CRMFLSRLVYCNTLKCQSSPQPPVYSLPPPVRPPPPPPPVPVSLPPPPTYCIHSPRPPPLFSPT
PTPILPPPPPNLRLQPLPPPSPIPCEKQPPPSPPPCEGQSPPLPTQNLPLPPPLSPTPPVYITPPV
PAPV
>XP_002984203.1 [Selaginella moellendorffii]
MAAMAAPHCLLGFLLLAFLILAASHQEHQESKNPAQRKLLQQPPAAAFPNPSLQAAYIALQ
AWKSRITGDPQGITATWIGLDVCRYRGVFCSPAPDDSCKEVVSGIDLNHGFLSGTLPEELGLL
TYLALFHINTNLFCGTLPGSMAALLLLYELDVSNNRFSGPFPLVTLAMPSLAYLDLRYNGFSG
ELPGALFDKHLDALFVNNNQFAAQVPWNLGSSTVSVANFANNRFSGGIPPSIGNMRRTLN
EIIFLGNRFDGCIPGEIGLLDQLNVFDVSYNSLGGGLPPSIAQMISLEQLDVAFNLLTGDVPAA
ICDLPNLENFTFAGNFFSGESPDCLALPSRGRIVDDRGNCIPNRPLQRPAQQCQAFLSQEPS
CFPLPPKSFFPNPPLPCSPPPSTPPY
>XP_017226212.1 [Daucus carota subsp. sativus]
MKLIPYLNSPLHSSLIILLFLTTFSHQTTLPPSYPPPQNPSLQNAYLALQAFKHAITSDPKGFTS
NWYGPYVCNYTGVYCAPAPDNPSHQTVAGIDLNHAEISGYLPEPLGLLKDLALFHINSNHF
YGTLPQSFSNLRLLYELDISNNAFSGEFPRVVFDLTSLKFLDIRFNQFQGEIPPNIFNMKLDALF
INNNNFQSNIPENIGNSPVSVFVLANNNLKGCFPSSITKMSNLNELILSNSGLTSCLPYDIGAL
TGLTVFDISFNTMTGPLPESMGRMKSLEQLNVAHNRFSGKVPASICYLPRLENFTYSDNYFY
GEDSMCLKLAEKDDRKNCLPARPLQRSVEECKAFYWKHVDCASSGCSARSHPPYGH
>XP_022034276.1 [Helianthus annuus]
MKALCCFLFISIISLNNAFATAITRRRLGPEPVDNVFEIRVDVRLHFPNFRLKKAYYALQEWKK
VITSDPKGMTKNWDGIEVCSYNGVFCAKAPDAPNLSTVAVVDLNHGDIEGKLPPHLGLISD
LSAFHINSNRFCGTIPNTFSRLTILNEFDISNNQFDGPFPNVVLDMPKLKYLDIRYNKFQGLLP
PQLFDKDLDAIFLNHNRFSSTIPENIGNSKASVIVFADNEFKGHIPKSIGQMPNLDEIIFANNQ
LSGCLPEELGLLHSTKVLDLSNNNFVGPIPRGIGNLKCIEMVDVAQNELIGTVVDVICTLPKLL
NFTFSDNYFDRLEGKCEKPMRPELVFDYRRNCLRGKPDQKDEGKCLPVVNRQIDCESLRSVL
DTNRRKPPKTKTRPTSVHGSITISFRH
>XP_021801086.1 [Prunus avium]
MKAAYFLVFFFLFSSFSSFSSALTDAEASFIARRQLLHFHAGHDLRGDHEYEVEVNLKFPHSRL
RRAYVALQAWKKAIYSDPIKTTENWVGANVCAYTGVFCAPALDDPQLEVVAGIDINHADIA
GYLPVELGMLSDIAVFHINTNRFCGIIPKSFARLTLLHELDVSNNRFVGSFPEVVLQIPNLKYLD
LRFNDFEGKLPPELFNKELDALFLNNNRFTSTIPDTLGNSPISVLVVANNHLEGCIPNSIGKMV
NNLNEAIFSHNKFAGCLPPEIGYLSNVTVFDVSSNTLSGLLSKTFKGLEKVEELNIAHNMLTGF
VPKSLCKLPSLGNFTFSYNYFNGEDQECVPPSRKDTLFDDTSNCLPNRPKQKSAKQCQVVV
NKPVDCGRAKCGGRPAPSKPSSPAPKEEPPKQQPPKEEPPKQQPPQEETPTLEPQPPKPELS
PEPW
>XP_010664614.1 [Vitis vinifera]
MQASGCFLLLSLLLSSPLSSFSLALSDEEASLIAHRQLLAIPKDGDLPNDFEYEVDLVVAFANA
RLRQAYIALQAWKHAMYSDPFNTTSNWEGANVCAYKGVFCAPALDNPKLSVVAGVDLNH

ADIAGYLPVELGLMTDLALFHINSNRFCGIIPKSFSRLTILHELDVSNNRFVGPFPAVVLSIPAL
RYLDLRYNEFEGELPPELFDKDLDALFVNHNRFTSNIPETLGHSRASVIVFAGNKFTGCIPRSIG
QMGNTLNEIIFASNDMSGCLPTEIGMLRNLTVFDAASNSFTGILPKSFNGLKQIEHLNVAHN
TLTGFVSDSICNLSSLSNFTFSYNYFKGEAKACEPPSRTDITLDDTSNCLPDRPKQKSAATCNP
VVSRPVDCSKAKCGGGASTPSPSPSSPPGDKASPPPHTTPSTTPKPSPTPSPKSTPSPSKPSPT
PSPPKPSPSSSPPKSTPAPFPPSPSPPKPSPAPSPKSSQTPSPKPSPSPSPPKSTPAPFPPSPSPP
KPSPA
>XP_020210238.1 [Cajanus cajan]
MASHSPKREGPYLNVLFLFVILISIINPSHQASSPTLPPLNPRLSKAYTALQAWKHSITSDPKN
FTSNWCGPHVCNYTGIYCAPALDDPYIYTVAGVDLKHAPISGSLPQELGLLTDLALFHINSNR
FCGSLPNTFDQLTLLHELDISNNQFSGPFPEPLLCLSSLKYLDIRFNNFHGNVPSRLFDLKLDA
LFINNNNFQFSLPQNFGNSPASVVVFANNDLKGCLPSSLVNMKGTLNEIIIMNSGLTGCLPP
EIGDLEKVTVFDVSFNKLVGELPESLGRMKKLEQLNVAHNMLSGTVPESVCMLPRLENFTYS
YNYFCTESPLCLKLKDKDDAKNCLPYRPLQRSREECEAFYAHPVQCSAFGCVTPPPPPPPPP
PPPPPPPPPPPPPPPPPPPPAAYYHYP
>XP_018625771.1 [Nicotiana tomentosiformis]
MQVYRCFFILLFSSILFSLSAALSDHEASLLARRQLSTLPENGKLPDNYEFEVHVNYTFPNSRFR
RAYIALKAWKEAVYSDPYKFTSNWKGPHVCKYKGVFCSPALDDPNVTVVAGIDLNHADIAG
YLPVELGLLTDAALFHLNSNRFCGIIPESFSRLRLMHELDLSNNRLVGPFPKVVLNMPNLKYL
DLRFNNFEGELPPQLFDKDLDALFLNDNRFVSTIPETIGNSSASVIVFANNKFHGCIPSSIGKM
SNLDEIVFRNNDLGGCLPVEVGLLKNITVFDVAGNSFSGILPKTLNALSHVEQLDLSHNTLTG
FVPENLCRLPSLKNFTFSFNYFNGEAKGCEPNVRKDVFFDDTNNCLRDRPKQKSQKQCQPI
VSKPIDCSKAKCGASSSKSLPHKEKKEKPQPQPPKPKPISPPTPKSPPTPKPSPKPQVFTPPPN
LKKASPPPPPLKSSLPPPVHSPPPPPVSSPPPQVFSPPPPVHSPPPPLRSPPPPVHSPPPPPVH
SSPPPPVHSPPPPPRLSPPPPFENVILPPNIGSIYASPPPPIFPGY
>XP_003568808.1 [Brachypodium distachyon]
MRTAAVALLLLLCCFAFAGGAAVWGQEGGRAMAAVEVDASWRFPSQRLRDAYVALQTW
KQRAIFSDPKGLTADWIGPGVCNYTGVFCAPLPSSPGELSVAGIDLNHGDIAGYLPPELGLLA
DLALLHLNSNRFCGLVPDALRRLRLLHELDLSNNRFVGAFPAVVLDLPALRFLDLRYNDFEG
GVPRELFDRPLDAIFLNHNRLRFSLPDNFGNSPVSVIVLADNHFGGCLPASLGNMSDTLNEIL
LINNGLSSCLPPEVGLLREVTVFDVSHNALAGPLPQELAGMRSVEQLDVAHNLLSGAVPDA
VCGLPRLKNFTFAYNFFTGEPPSCARVVPADGGDRRNCLPNRPAQRVPQQCAAFYARPPV
DCAAFQCKPFVPPPPPPPPAYPGPLPPVYPMPYASPPPPAHYR
>XP_008371522.1 [Malus domestica]
MQAYGCFLVSFFILALFSFSSSALTDAEASFLAHRQLVSLPEGGDIPDNYEFEVELDLKFPNTRL
RRAYIGLQALKKAVYSDPLKTTENWVGENVCAYNGVFCAPALDDPELEVVAGIDINHADIA
GHLPAELGLLTDMALFHINSNRFCGIIPKSFRRLTLLHEFDVSNNRFVGSFPDVVLEIPNLKYL
DLRFNDFEGKLPPELFNKELDALFLNDNRFTSTIPENLGNSPVSVLVVANNQLEGCIPNSIGK
MVKTLNEVVFSNNKFTGCLPPEIGQLANMTVFDISSNTFSGIMSKTFKGLEKVEELDIAHNML
TGFVPNSICTLPNLGNFTFSYNYFNGETQKCVPGSRKDVVFDDVSNCLPDRPEQKSAKECH
VVVSKPVDCSKAKCGGGHGLLKPSQPLVEKPKTPEPEQPKQPPPQPKPQPPKPSPELVQTPH
TPKPPKEEQPMEQPPKEEQPKEQTCQRGATPGTTS
>XP_023769647.1 [Lactuca sativa]
MKPICCFLFLSIFIFIFSSPSSSIPLNNAFTAAITRRHLSDTPKEIDPPPDDDLACEIETDARLNFP
NPRLKKAYCVLQQWKKVIYSDPENMLSNWEGADVCSYKGVFCEKALDDPNVVTVAGIDLN

HGDIAGQLICLLGLLTDLAIFHINSNRFCGTIPTSFSRLTILHEFDISNNRFVGPFPNVVLEMPKL
KYLDIRFNDFEGGLPPELFDKDLDAIFLNDNRFTSTIPENIGNSNASVIVFADNDFKGCIPKSIG
QMTSLDEVIFANNELSGCVPEELTMLENIKVLDLSNNNFVGTLPNGFERLKNVESIDIGRNELI
GKVVESVCTLPKLMNFSFSKNFFDGLEPKCEKPMKAELVLDDRENCLPKKPNQKTEQKCSPV
VNQPIDCKSLGCKKPSRTSDEESKKRKPRRRSPPPPPPVQSPPPPPPVQSPPPPVFSPPPPVP
SPPPPTPSPPPPSPPPPVLSPPPPPPPVPSPPPPSFNDVDIPQYIGSRYPSPPPPVHPGY
>XP_008458675.1 [Cucumis melo]
MGSHVPIQRQPFHHGFSSQKASSPCLLSYYSFLFFSLPSFSVSISEAEASYLTRRQLLALKEHDE
LPDDFKYQLDIADTFPNERLKMAYVALQAWKLAIYSDPQNMTANWVGADVCSYTGVFCA
PALDNPNIEVVAGIDLNHGDIAGYLPPELGLLTDLALFHINSNRFCGIIPSSFSKLVLMFEFDVS
NNRFVGHFPLVVLEWPGARYLDLRYNDFEGEIPSTLFTKEFDAIFLNNNRFNSLIPETIGNSTV
SVVSFANNEFHGCIPGTIGQMSNLNQILFLGNKLSGCFPPEIGNLINLTVFDVSSNGFIGQLPE
SLSGLQNLEILDVSNNELRGSVSGGLCKLPKLANFTFSFNYFDGQDTTCVASKGSEKVFNDG
QNCLANRPVQKDANKCSTVLKKSMECGNCGGGSSSPGVPSTPEDPYDQSPSPRYRSPPPP
VVTPPSPSETSVPSSPPSPVESSPPTSTSSSPPTIETPSSSPLPPPPVYSPPPPSVRSPPPPVHSPP
PPVHSPPPPVHSPPPPVHSPPPPVHSPPPPVHSPPPPVHSPPPPVHSPPPPVHSPPPPVHSP
PPPIHSPPPPVYSPPPPVHSPPPPVYSPPPPIHSPPPPVYSPPPPVYSPPPPPVYSPPPPVQSPP
PPTASPPPSPSINSSPPPSFEDVILPPNIGFEYASPPPPLFPGY
>XP_022573025.1 [Brassica napus]
MDKPLGTFFILLLISPIVVATINEETSFPENAHLTNNLDQKCVDIIKVDPSLKFENDRLKRAYIAL
QAWKKAIYSDPFKTTKNWVGPDVCSYNGVYCAEALDDPSLKVVAGVDLNHADIAGHLPAE
LGLITDLAMFHINSNRFCGIIPKSLSKLALMYEFDVSNNRFVGPFPEVSLSWPSLKFLDLRYNE
FEGCLPSEIFDKNLDAIFLNNNRFESVIPDTIGKSAASIVTFANNKFSGCIPRSIGQMKNLNEVI
FTGNNLTGCFPNEIGSLNNVTVFDASNNGFIGSLPLTLSSLSRVEQLDLSNNKLTGSVVDTFC
KLPNLERFKFSYNYFNGEAESCVHGKNNGKQFDDRSNCLKNRPDQKSDQNTLQVRIREVQ
QKQSR
>XP_006300291.2 [Capsella rubella]
YAHIYKQPWVFSKVLFKAMNKPTISLPPFGCFFLFFSLFFFFSLFSSVVLALTDAEASFIPQRQLL
TLPENGELPDDIEYEVDLKVTFANHRLKRAYIALQAWKKAVYSDPFNTTGNWHGPHVCGYT
GVFCASALDDPNVAVVAGVDLNGADIAGHLPAELGLMTDVAMFHLNSNRFCGIIPKSLEKL
RLMHEFDVSNNRFVGPFPSVVLSWPAVKYIDVRYNDFEGQVPPELFKKDLDAIFLNNNRFTS
TIPDSIGESSASVVTFAHNKFNGCIPKTIGNMKNLNEIIFKDNNLGGCFPSEIGKLANVNVFD
ASMNSFTGVLPPSFVGLTGVEEFDISGNKLTGFVPENICKLPKLVNLTYAFNYFNGQGDLCIP
GSRTEIALDDTRNCLPDRPKQRSARECAIVISRPVDCSKDKCAGGSSHVAPSRSTSPVPTRPV
HKPQTPKESPPVPTRPVHKPQPPKESPHVPTRPVHKPQPPKESPHVPTRPVHKPQPPKESLL
PTTTSSKVTTTTTNTNTDSRSTTTK
>XP_007200697.2 [Prunus persica]
MAPSLITNKPSGGCLLFFLLFSSFSALSFALTDAEVAFIAHRQLLALPEGGDLPENYVVQVDAK
LNFENDRLKNAYVALQALKKAIFSDPFGFTVNWEGEDVCSYKGVFCAPALDNEKLTVVAGL
DLNHADIAGHLPVEMGLLTDAALIHLNSNRFCGIIPRSFRRLILMHEFDVSNNRFVGSFPVVV
LEWPSCKYLDLRYNDFEGELPPELFHKEHDALFLNHNRFTSIIPDTLGHSTVSVVNFGFNNFT
GCIPRSVGNMKNLNEIIFMNNQLGGCFPPEIGNLGNLQVLDVSNNVFIGSFPKTFLELRSIEEL
AVGYNRLTGFVTEKICSLPKLANFTFAYNYFSGEAQKCMPNPKLEVNFDDTGNCMPGRPKQ
KNTKTCFPVVTKPVDCSKHCGGGSSTPDKPKTPRPQPPQTPKTEKPPTPKAEPPPTPKAEPPP
TPKAKPPPTPKAEPPPTPKAEPPPTPKAEPPPTPKAEPPPTPKAEPPPTPKAEPPPTPKAEPPPT

PKAEPPPTPKAEPPPTPKAEPPPTPKAEPPPTPKAEPPPTPKAEPPPTPKAEPPETPEAEPPQTP
EAEPPQTPEVEPPPTPKAEPPPTPKAEPPPTPKAEPPPTPKSEPPPTPKAEPPPTPKAEPPPTPN
LSCVYC
>XP_020099514.1 [Ananas comosus]
MEASPLSSLPFLLLLLLLSAAGLLSLTSALSDAETVFIARRQLLTFHEHSGDLPEDFEFGVQIDTP
FPNQRLRRAYIALQAWKHAIYSDPSNFTGNWSGADVCSYAGVFCSPALDDPSLTVVSGVDL
NGADIAGYLPAELGLLTDLALFHINSNRFCGIIPKSFSRLVLMYELDVSNNLFVGPFPVVVLGL
PALRYLDLRFNNFEGALPPELFDKELDAIFLNDNRFSSYIPENLGNSTASVIVAANNKLQGCIP
TSVGKMTSTLNELVLLNNELTGCLPLEFGLLTNVTVFDASFNALTGVLARSLAGLAKVEQLDV
SHNVLTGVVPEGLCRLPRLANFSFSYNYFKGEAEACTPVPRSEVLFDDKSNCLGSSRPNQKS
AKECAPVISRPVDCSRSKCSSSPSRSAPSPKPLTPSHKPSPKPIQPPKPTPKPSPKTVAPSPKPT
PTPSPKALATTPTSELLTTTTTVALLLSAAARMMTSTTTHFASSTPHLTTSSSTT
>XP_020579786.1 [Phalaenopsis equestris]
MPFANSSSTFLLFLCLLISTLPFHSLSQFSFPNPRLEKAYIALQAWKHAITSDPKNLTGNWCGP
HVCNYTGIFCAPAPDDPSITTVAGIDLNHGSISGTLPDHLSLLSDLALFHLNSNRFSGMLPSSL
CDLRRLFELDVSNNHLSGPFPDFVLRLPELRFLDLRFNAFLGDVPAEVLNGGGSAAKLDAFFI
NDNNFKFSLPANLGNSKASVIVLANNRIEGTLPASIGGMGKTLNQLVILNTGLAGCIPPEIGN
LKRLTVFDVSFNALVGPLPASMGEMKKLEQLDVAHNFLSGEIPEGICELPRLKNFTYSYNYFC
GEPQRCLKVRRQDDRRNCIPWRPQQRSPAECAAFLSRPVHCDAYHY
>XP_004960846.1 [Setaria italica]
MRKALLLLLFVLCCLATAVEAVGEGAAQAGAELAVVVDPSWRFPSQRLRDAYVALQTWKQ
QAIFSDPRNFTADWVGPGVCSYRGVFCAPLPQGEPGAGELAVAGIDLNHGDIAGYLPSELG
LLTDLALLHLNSNRFCGLVPAALRRLRLLVELDLSNNRLVGAFPAVVLDLPALKFLDLRFNDFE
GPIPRELFDRPLDAIFLNHNRLRSPLPDNFGNSPASVIVLADNSFGGCLPASLGNMSGTLNEI
LLINNGLDSCVPPEVGLLREVTVFDVSFNALVGPLPQQVAGMRKVEQLDVVHNHLSGAIPE
AICALPRLKNFTVSDNYFTGEPPSCARVVPPDGDRRNCLPNRPAQRTPQQCAAFYSLPPVD
CAAFQCKPFVPVPPLPPPPPVYPGPLPPVYPMPYASPPPPSHYR
>XP_011656354.1 [Cucumis sativus]
MFNTATTHFTMASLHRKQAHHACFLIILFLFFSLPSFSVSLSEAEASYLTRRRLLTLKNHYELPD
DFQYELDIADTFPNERLKKAYRALQAWKLAVYSDPQNMTANWVGADVCSYTGVFCAPAL
DDPKIEVVAGIDLNHGDIAGHLPPELGLLTDLALFHINSNRFCGIIPSSFSNLVLMFEFDVSNN
RFVGHFPLVVLEWPSAKYLDLRYNDFEGEIPSTLFTKEFDAIFLNNNRFNSLIPETIGNSTVSVV
SFANNEFHGCIPSTIGQMSNLNQILFLGNKLSGCFPPEIGNLVNLTVFDVSNNDFIGQLPESL
SSLQNLEIMDVSNNELRGSVSGGLCKLPKLANFTFSFNYFDGEDAACATSKGSEKLFDDSQN
CLANRPMQKDANKCSTVLKKSVDCGNCGGGSSSPGVPSTFEDPYDQSPSPRYRSPPPPVVT
PPSPSPSETSVPSSPPSPIESSPPISTPSPPPPVNSPPPPVHSPPPPVHSPPPPVHSPPPPVHSPPP
PVHSPPPPVYSPPPPIHSPPPPVYSPPPPVHSPPPPVYSPPPPVHSPPPPVYSPPPPPVQSPPP
PTASPPPPPSHDSSPPPSFEDVILPPNIGFQYASPPPPLFPGY
>ClPEX [Citrullus lanatus]
MASFHRKQANHACFLLLFLFFSLPSLSFSISKDEASYLTHRQLLALKEHDQLPDDYKYQLDIPD
TFPNERLKKAYVALQAWKLAIYSDPQNMTANWVGADVCSYTGIYCTTALDDSDMEVVAGI
DLNHGDIAGYLPPEIGLITDLALFHINSNRFCGIIPSSFSKLVLMFEFDVSNNRFVGHFPHVVLE
WPTAKFLDLRYNDFEGEVPSTLFTKEFDAIFLNNNRFTSLIPDTIGNSTVSVVSFANNEFHGCI
PSTIGQMSNLNQILFIGNKLDGCFPPEIGNLVNLTVFDASANGFIGHLPETLSGLQNLEIMDV
SNNELRGSVPGGLCKLPKLANFTFSFNYFDGEDATCVGTKGSEKLFDDAQNCLANQPEQKD

ANKCNVVLKKSVDCGCSGGGSGSPGVPMTPEDPFDQSPSPRYRSPPPPVISPPESIDTLVPE
VETPSAPSLSPISAPFSPPNLETPNPSPVDSLEPPVHSPPPPVHSPPPPVHSPPPPVHSPPPPV
HSPPPPVHSPPPPVHSPPPPVHSPPPPIHSPPPPVHSPPPPPIPSPPPPVHSPPPPVHSPPPPV
HSPPPPVHSPPPPVHSPPPPVYSPPPPVHSPPPPIHSPPPPVHSPPPPVHSPPPPVYSPPPPV
HSPPPPVYSPPPPVQSPPPPTASPPPPPPDSSPPPTFEDVILPPNIGFQYTSPPPPQFPGY
>ClPEX [Citrullus lanatus]
MASFHRKQANHACFLLLFLFFSLPSLSFSISKDEASYLTHRQLLALKEHDQLPDDYKYQLDIPD
TFPNERLKKAYVALQAWKLAIYSDPQNMTANWVGADVCSYTGIYCTTALDDSDMEVVAGI
DLNHGDIAGYLPPEIGLITDLALFHINSNRFCGIIPSSFSKLVLMFEFDVSNNRFVGHFPHVVLE
WPTAKFLDLRYNDFEGEVPSTLFTKEFDAIFLNNNRFTSLIPDTIGNSTVSVVSFANNEFHGCI
PSTIGQMSNLNQILFIGNKLDGCFPPEIGNLVNLTVFDASANGFIGHLPETLSGLQNLEIMDV
SNNELRGSVPGGLCKLPKLANFTFSFNYFDGEDATCVGTKGSEKLFDDAQNCLANQPEQKD
ANKCNVVLKKSVDCGCSGGGSGSPGVPMTPEDPFDQSPSPRYRSPPPPVISPPESIDTLVPE
VETPSAPSLSPISAPFSPPNLETPNPSPVDSLEPPVHSPPPPVHSPPPPVHSPPPPVHSPPPPV
HSPPPPVHSPPPPVHSPPPPVHSPPPPIHSPPPPVHSPPPPPIPSPPPPVHSPPPPVHSPPPPV
HSPPPPVHSPPPPVHSPPPPVYSPPPPVHSPPPPIHSPPPPVHSPPPPVHSPPPPVYSPPPPV
HSPPPPVYSPPPPVQSPPPPTASPPPPPPDSSPPPTFEDVILPPNIGFQYTSPPPPQFPGY
